# Supplementary material for: Effect of Interactions between Phosphorus and Light Intensity on Metabolite Compositions in Tea Cultivar Longjing43
Source: Int J Mol Sci. 2022 Dec 2;23(23):15194. doi: 10.3390/ijms232315194 (PMC9740319; doi:10.3390/ijms232315194)
Supplement: Supplementary file 1 [file ijms-23-15194-s001.zip › ijms-2041895-supplementary figures.pdf]

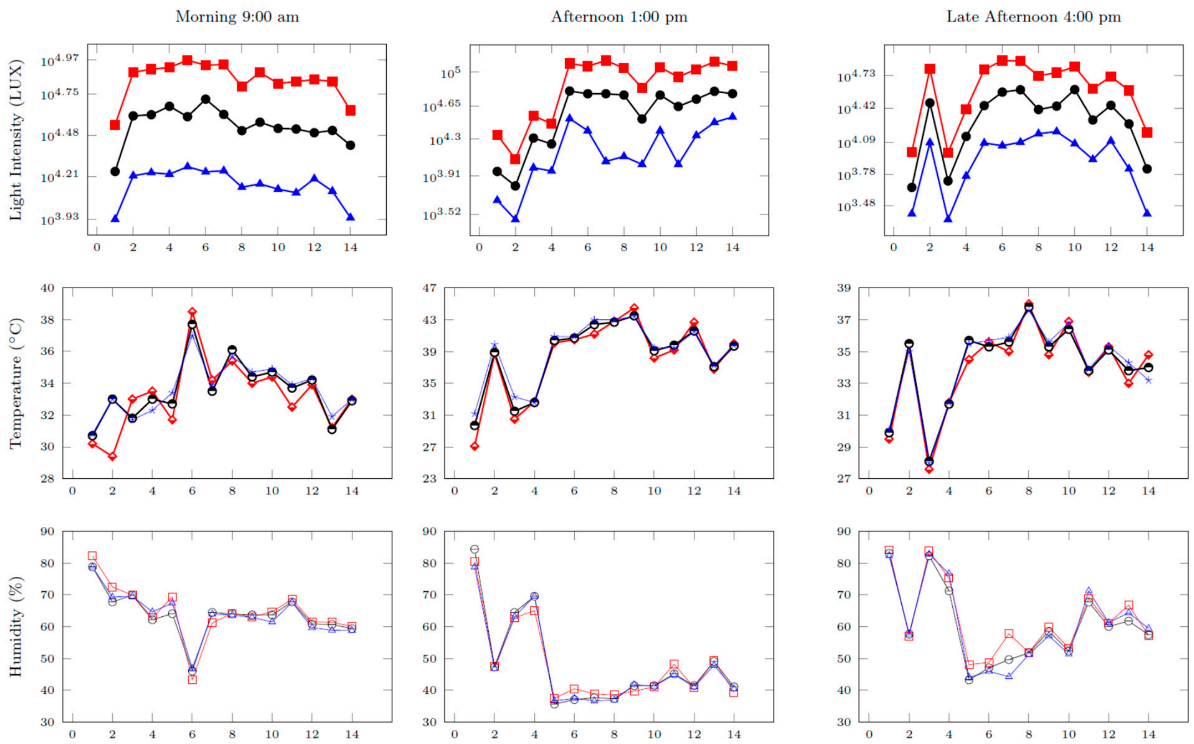

Parameters	Legend	Morning	Afternoon	Late Afternoon	Average
Light Intensity	FL	$7.2 \times 10^4 \pm 1.6 \times 10^4$	$8.6 \times 10^4 \pm 4.2 \times 10^4$	$4.6 \times 10^4 \pm 2.2 \times 10^4$	$6.8 \times 10^4 \pm 3.3 \times 10^4$
	ML	$3.6 \times 10^4 \pm 8.3 \times 10^3$	$4.3 \times 10^4 \pm 2.0 \times 10^4$	$2.3 \times 10^4 \pm 1.2 \times 10^4$	$3.4 \times 10^4 \pm 1.7 \times 10^4$
	LL	$1.5 \times 10^4 \pm 3.1 \times 10^3$	$1.7 \times 10^4 \pm 1.0 \times 10^4$	$9.4 \times 10^3 \pm 4.5 \times 10^3$	$1.4 \times 10^4 \pm 7.4 \times 10^3$
Temperature	FL	33.21±2.19	38.21±4.77	34±2.68	35.14±4.05
	ML	33.54±1.8	38.55±4.16	34.14±2.52	35.63±3.79
	LL	33.61±1.61	38.99±3.82	34.2±2.55	35.38±3.65
Humidity	FL	64.64±8.24	47.88±12.38	62.44±11.4	58.32±13.13
	ML	63.82±6.81	48.12±13.98	60.32±11.68	57.42±13.09
	LL	63.85±6.95	47.42±12.89	60.74±12.81	57.34±13.30

Figure S1. Light intensity, temperature and humidity above the canopy of the tea plants exposed under full light (FL), medium light (ML) and low light (LL) intensity at morning, afternoon and late afternoon during sample collection time (14 weeks).

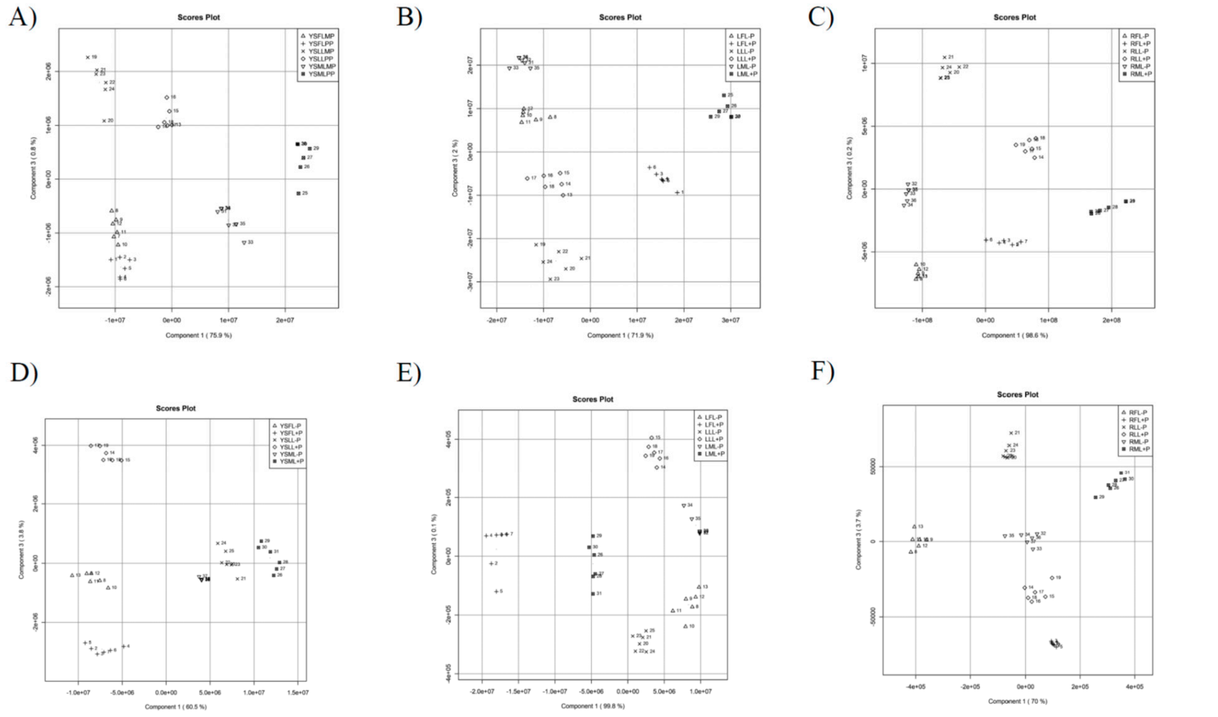

Figure S2. PCA analysis of Primary Metabolites (A-C) and Secondary metabolites (D-F) captured by GC×GC-TOF/MS & UPLC-Q-TOF/MS in young shoots (A,D), leaves (B,E) and root (C,F) of Longjing-43 cultivar due to light (FL, ML & LL) and P (+P & -P) effects.

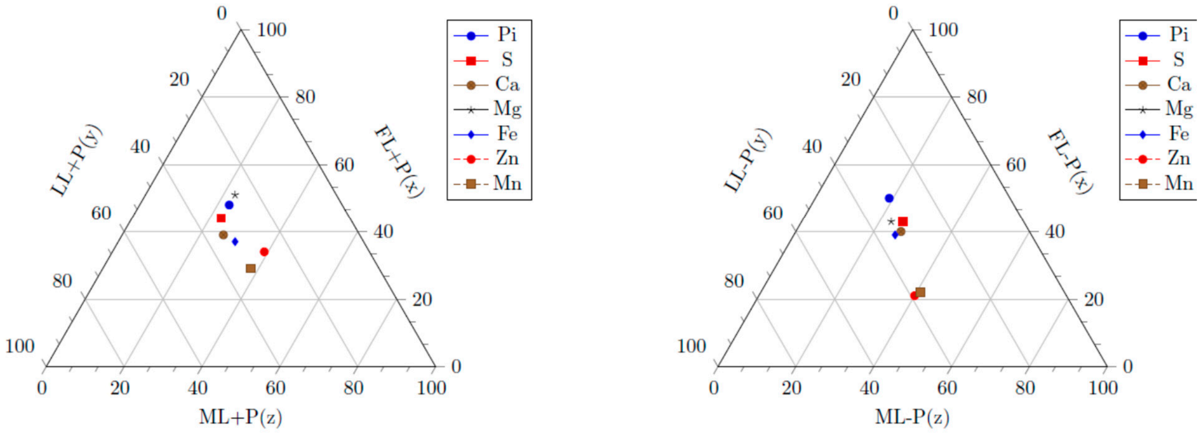

Figure S3. Ternary Graph of young shoots exposed to Full light (FL), Medium Light (ML) and Low light (LL) under P sufficient (+P) and P deficient (-P) conditions.

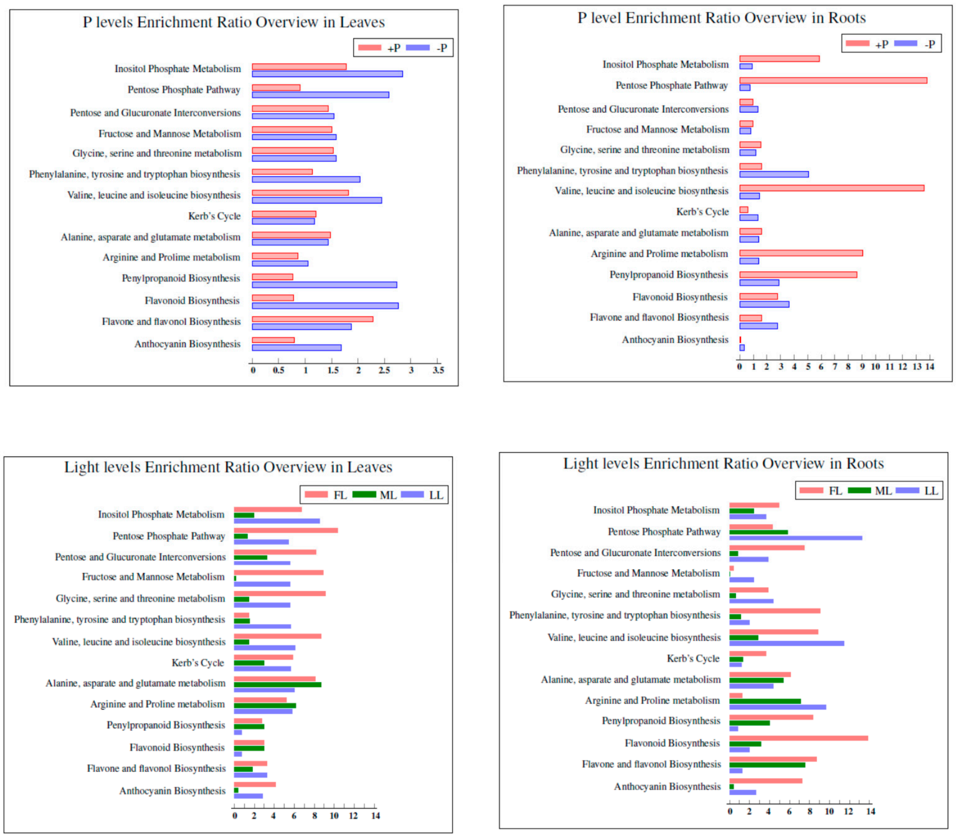

Figure S4. Enrichment ratio due to P levels and light regime in leaves and roots.

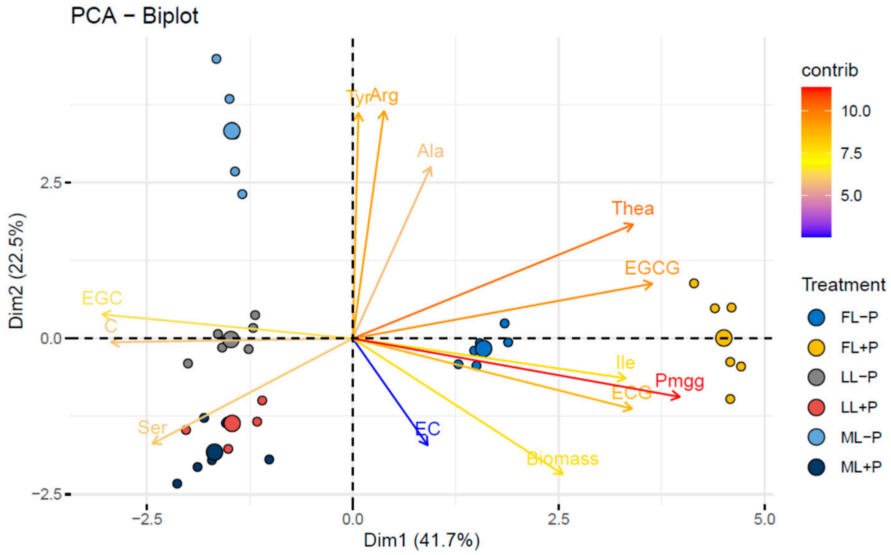

Figure S5. A single bi–plot for the dataset combines both samples and treatments of leaves to the principal components.

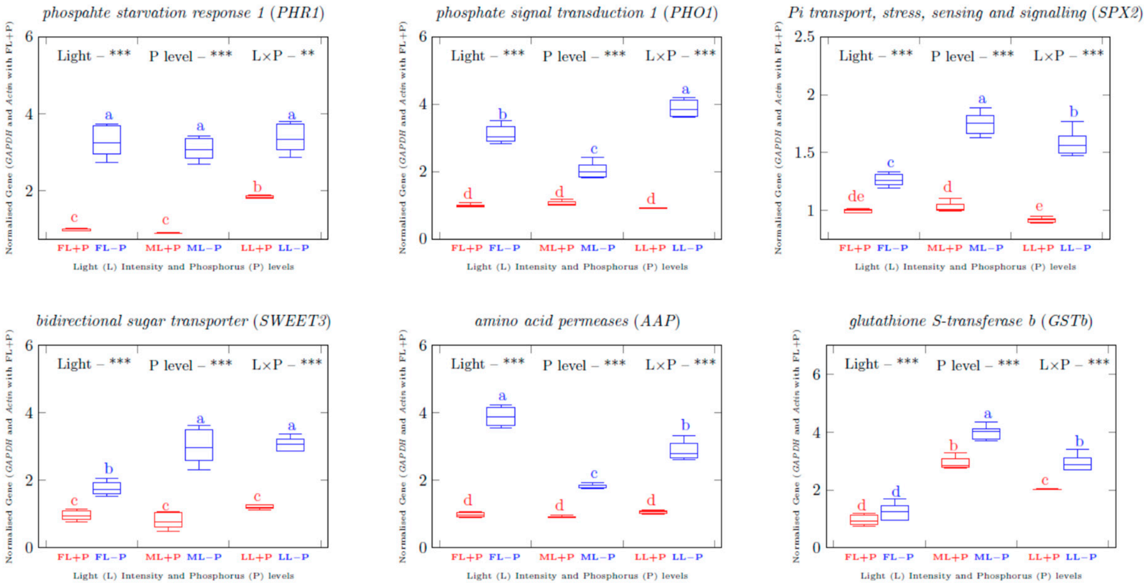

Figure S6. qRT–PCR analyzed transcription and transporter gene transcripts normalized (*GAPDH* and *Actin*) with sample full light with +P. '***' 0.001 '**' 0.01 '*' 0.05 = significant differences, ns = non-significant differences, between light and P interaction.

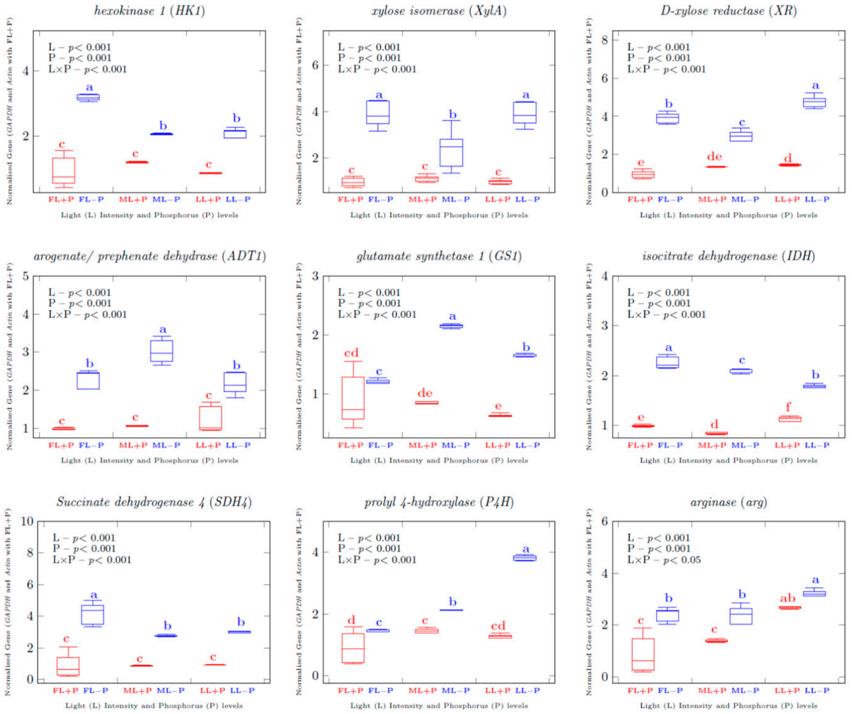

Figure S7. qRT–PCR analyzed primary metabolites pathway gene transcripts normalized (*GAPDH* and *Actin*) with sample full light with +P. '***' 0.001 '**' 0.01 '*' 0.05 = significant differences, ns = non-significant differences, between light and P interaction.

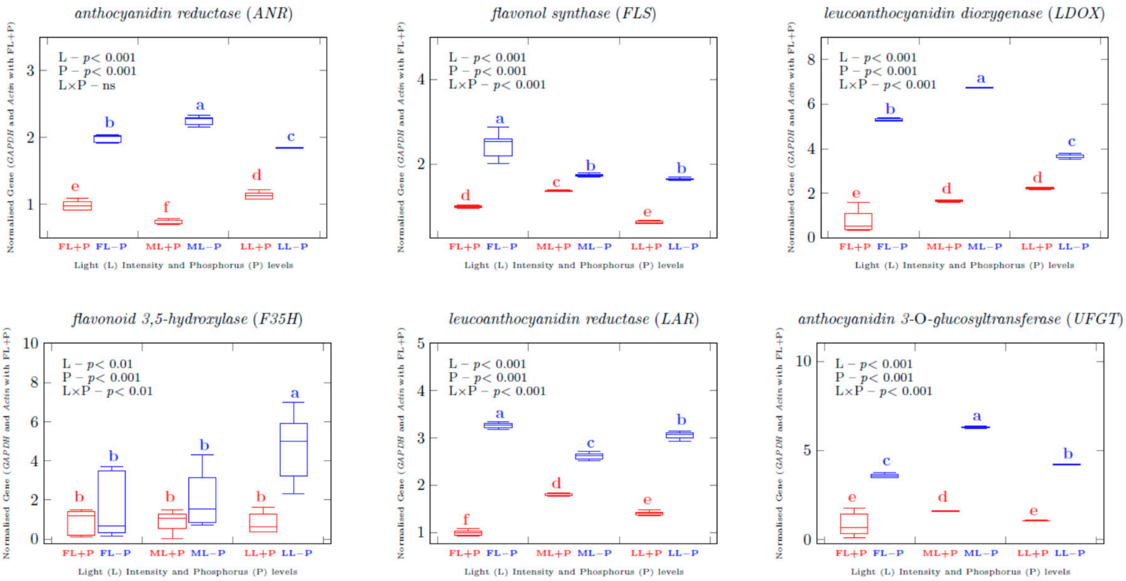

Figure S8. qRT–PCR analyzed secondary metabolites pathway gene transcripts normalized (*GAPDH* and *Actin*) with sample full light with +P. '***' 0.001 '**' 0.01 '*' 0.05 = significant differences, ns = non-significant differences, between light and P interaction.

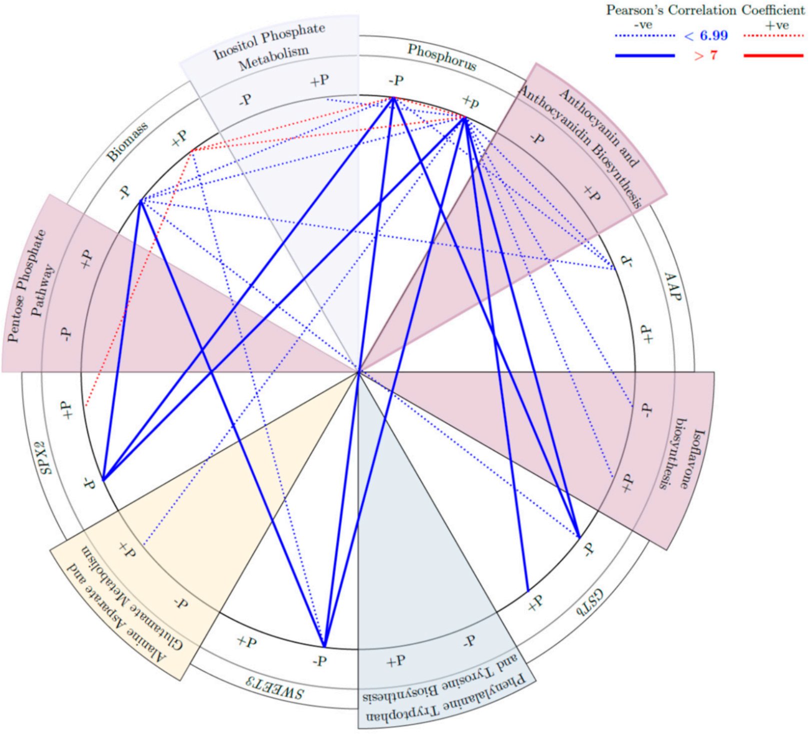

Figure S9. Circular representation of correlation between pathways with phosphorus and biomass under different light regimes.
