# Supplementary material for: Effect of Interactions between Phosphorus and Light Intensity on Metabolite Compositions in Tea Cultivar Longjing43
Source: Int J Mol Sci. 2022 Dec 2;23(23):15194. doi: 10.3390/ijms232315194 (PMC9740319; doi:10.3390/ijms232315194)
Supplement: Supplementary file 1 [file ijms-23-15194-s001.zip › ijms-2041895-supplementary tables.pdf]

Table S1. Primer sequences for Longjing-43 cultivar for qRT-PCR analysis.

Pathway	Gene Identifier	Forward primer (5'>3')	Reverse primer (5'>3')	References
PHR1 (*phospahte starvation response 1*)	CL15435Contig1	ATCTGCACAATGAACAGCACCAT	GCTCCAAAGAGGCTTAGGATAGA	KC et al. (2018)
PHO1 (*phosphate signal transduction 1*)	CL27009Contig1	TCGAGTCCTGGTAGGAAGAACCT	GTTGATTATTGGCAACTCAAGAAAG	KC et al. (2018)
SPX2 (*Pi transport, stress, sensing and signalling*)	CL117Contig2	CAGGGTAGCAGAGGCTCGGGATT	AGCACCGCTTCGCTTGTCGTATT	KC et al. (2018)
SWEET3 (*bidirectional sugar transporter*)	CL73507Contig1	GGCTGCCAACCAGATTAGGA	TCCTCTGGTGGTCGTGAAGA	
AAP (*amino acid permeases*)	CL10295Contig1	TTGAGTCTCAGTCACCGTGC	TCGGTCTTGGAATCGGCAAA	
GSTb (*glutathione S-transferase b*)	CL12598Contig1	AGTGGTCGGTTGGATCCCTA	ATGGAGGCAAGGGAAGTTCG	
Actin		AGAAGTCCTGTTCCAGCCGTCTT	CGAACCACCACTGAGCACTATGTT	Li et al. (2016)
GAPDH		GTGGTTCTTGTGAGTTTCTGGA	ACCCTTCTCTTCAAGGGCAAT	Xiong et al (2013)

References

KC, S.; Liu, M.; Zhang, Q.; Fan, K.; Shi, Y.; Ruan, J. Metabolic Changes of Amino Acids and Flavonoids in Tea Plants in Response to Inorganic Phosphate Limitation. Int. J. Mol. Sci. 2018, 19, 3683. https://doi.org/10.3390/ijms19113683

Li et al. (2016) Li MY, Wang F, Jiang Q, Wang GL, Tian C, Xiong AS. Validation and comparison of reference genes for qPCR normalization of celery (*Apium graveolens*) at different development stages. Frontiers in Plant Science. 2016b;7:313. doi: 10.3389/fpls.2016.00313.

Xiong, L., Li, J., Li, Y., Yuan, L., Liu, S., Huang, J., Liu, Z. Dynamic changes in catechin levels and catechin biosynthesis-related gene expression in albino tea plants (Camellia sinensis L.). Plant Physiologyogy and Biochemistry, 2013, 71: 132-143.

Table S2. Primary metabolites related gene transcripts primer sequences for the of Longjing-43 cultivar of selected pathways for qRT-PCR analysis.

Pathway	Gene, reaction and Enzymatic activity	Gene Identifier	Forward primer (5'>3')	Reverse primer (5'>3')	References
Fructose and Mannose metabolism	*hexokinase 1* (2.7.1.1, *HK1*)	CL42645Contig1	CCCGTGTCGCAACTATGTCA	CGGCTCTCTTCGGTGAAACT	KC et al. (2018)
Inositol phosphate metabolism	*inositol-phosphate phosphatase 1* (3.1.3.25, *IMPL1*)	CL37584Contig1	TCGATTTCGGGTGTCTTGTAGG	TGATGACATGGTCGCATCCAA	KC et al. (2018)
Pentose phosphate pathway	*ribose 5-phosphate isomerase A* (5.3.1.6, *R5PIA*)	CL76822Contig1	GACTCGGAGTGATGGCAAGT	AATGGTAGGCACATGGAGGC	KC et al. (2018)
	ribokinase synthase (2.7.1.15, *RBKS*)	CL66999Contig1	ACAACCCCACAACCCTCAAG	AGACTGGCCAAACACTTGCT	KC et al. (2018)
Pentose and Glucuronate Interconversions	*xylose isomerase* (5.3.1.5, *XylA*)	CL2313Contig1	GTCGGATTGGCTTCGCTTTG	AGGATTAGTAGCAGCACCGC	KC et al. (2018)
	D-xylose reductase (1.1.1.15, *XR*)	CL23910Contig1	CCGATAGACTTCACCAGCCC	TCACGTTCCGATTGAAGCGA	KC et al. (2018)
Phenylalanine,Tyrosine and Tryptophan biosynthesis	*arogenate/ prephenate dehydrase* (4.2.1.51/4.2.1.91, *ADT1*)	CL9637Contig1	CAACTGGTCGTTTGTCCGTAATG	CTTGCTCAGACTTCCTAAGAGGC	KC et al. (2018)
Alanine, Asparate and Glutamate Metabolism	*glutamine synthetase 1* (6.3.1.2, *GS1*)	CL66758Contig1	TCCAAGCTTTTCGATGGCCT	GTGGAGAGCCAATCCCAACA	KC et al. (2018)
Kerb's cycle	*isocitrate dehydrogenase* (1.1.1.42, *IDH*)	CL31117Contig1	TACGCGTGCCTAATCAGGAG	AGGTGTTGAACCCAGTGGTG	KC et al. (2018)
	Succinate dehydrogenase 4 (1.3.5.1, *SDH4*)	CL33432Contig1	CCTACGACAACCGCATCGT	CCCCACATTCGAAGACCCTT	KC et al. (2018)
Arginine and Proline biosynthesis	*prolyl 4-hydroxylase* (1.14.11.2, *P4H*)	CL27105Contig1	CGTCGCCTTTCTTCGGTTTC	TCTGATGTGACCAAAGGCGG	KC et al. (2018)

Continue on the next page

Table S2. Primary metabolites related gene transcripts primer sequences for the of Longjing-43 cultivar of selected pathways for qRT-PCR analysis (*cont.*).

Pathway	Gene, reaction and Enzymatic activity	Gene Identifier	Forward primer (5'>3')	Reverse primer (5'>3')	References
	arginase (3.5.3.1, *arg*)	CL40328Contig1	CCGCTCTCTTCATCAGCTCC	ACCTCCTAAAGCACGCACAA	KC et al. (2018)
Isoavone Biosynthesis	*flavonol 3-O glycosyltransferase* (2.4.1.91, *UGT78D2*)	CL89596Contig1	CAGGGATGGAACATGTCGCT	AAATTACCCACCGCCGAGTT	KC et al. (2018)
	flavonol-3-O-glucoside L-rhamnosyltransferase (2.4.1.159, *UGT78D1*)	CL25776Contig1	AACCATGGGTCCCAATAGCG	ACAGAATAACTCGCCGCACA	KC et al. (2018)
Flavanones Biosynthesis	*flavonoid 3′,5′-hydroxylase* (1.14.14.81, *F3′ 5′ H*)	CL54192Contig1	CATCTCCGCTAGTGCCCATT	ATGATGAGAGGCTCAGCGTG	KC et al. (2018)
Anthocyanidins Biosynthesis	*leucoanthocyanidin reductase* (1.17.1.3, *LAR*)	CL77394Contig1	CTGCAGCAAGGAGGTCATCT	TCAGGGGTGCCCTACACTTA	KC et al. (2018)
	*anthocyanidin 3-*O*-glucosyltransferase* (2.4.1.115, *UFGT*)	CL89161Contig1	CATAGGCTCCCGAAGCAGAG	GAATCACCGCCAGTTTACGC	KC et al. (2018)
Flavones Biosynthesis	*glucosyltransferases* (2.1.4.81, *UGT75L12*)	CL90073Contig1	AGTTCTTTCGCACCCGTCAT	TCTTCCACCAGCTTCGCATT	KC et al. (2018)
Anthocyanin and Anthocyanidins Biosynthesis	*leucoanthocyanidin dioxygenase* (1.4.11.19, *LDOX*)	CL59191Contig1	CAACCGGCTTCCATACCCTT	TTGGCGATGACAGTGCTCTT	KC et al. (2018)
	anthocyanidin reductase (1.3.1.77, *ANR*)	CL2560Contig1	ACTTGTGTTGTTGGCGATCC	ACAAGGCTAGGGCTAAAGCA	KC et al. (2018)

References

KC, S.; Liu, M.; Zhang, Q.; Fan, K.; Shi, Y.; Ruan, J. Metabolic Changes of Amino Acids and Flavonoids in Tea Plants in Response to Inorganic Phosphate Limitation. Int. J. Mol. Sci. 2018, 19, 3683. https://doi.org/10.3390/ijms19113683

Table S3. Secondary metabolites related gene transcripts primer sequences for the of Longjing-43 cultivar of selected pathways for qRT-PCR analysis.

Pathway	Gene, reaction and Enzymatic activity	Gene Identifier	Forward primer (5'>3')	Reverse primer (5'>3')	References
Isoflavone Biosynthesis	*flavonol 3-O glycosyltransferase* (2.4.1.91, *UGT78D2*)	CL89596Contig1	CAGGGATGGAACATGTCGCT	AAATTACCCACCGCCGAGTT	KC et al. (2018)
	flavonol-3-O-glucoside L-rhamnosyltransferase (2.4.1.159, *UGT78D1*)	CL25776Contig1	AACCATGGGTCCCAATAGCG	ACAGAATAACTCGCCGCACA	KC et al. (2018)
Anthocyanidin and Anthocyanin Biosynthesis	*anthocyanidin reductase* (1.3.1.77, *ANR*)	CL2560Contig1	ACTTGTGTTGTTGGCGATCC	ACAAGGCTAGGGCTAAAGCA	KC et al. (2018)
	flavonol synthase (1.14.20.6, *FLS*)	CL18757Contig1	CTCGTTGATCGGACGGCTAA	ACGACAAACACAGCCCAAGA	
	leucoanthocyanidin dioxygenase (1.4.11.19, *LDOX*)	CL59191Contig1	CAACCGGCTTCCATACCCTT	TTGGCGATGACAGTGCTCTT	KC et al. (2018)
	flavonoid 3′,5′-hydroxylase (1.14.14.81, *F3′ 5′ H)*	CL54192Contig1	CATCTCCGCTAGTGCCCATT	ATGATGAGAGGCTCAGCGTG	KC et al. (2018)
	leucoanthocyanidin reductase (1.17.1.3, *LAR*)	CL77394Contig1	CTGCAGCAAGGAGGTCATCT	TCAGGGGTGCCCTACACTTA	KC et al. (2018)
	anthocyanidin 3-O-*glucosyltransferase* (2.4.1.115, *UFGT*)	CL89161Contig1	CATAGGCTCCCGAAGCAGAG	GAATCACCGCCAGTTTACGC	KC et al. (2018)
Flavones Biosynthesis	*glucosyltransferases* (2.1.4.81, *UGT75L12*)	CL90073Contig1	AGTTCTTTCGCACCCGTCAT	TCTTCCACCAGCTTCGCATT	KC et al. (2018)

References

KC, S.; Liu, M.; Zhang, Q.; Fan, K.; Shi, Y.; Ruan, J. Metabolic Changes of Amino Acids and Flavonoids in Tea Plants in Response to Inorganic Phosphate Limitation. Int. J. Mol. Sci. 2018, 19, 3683. https://doi.org/10.3390/ijms19113683

Table S4. Concentrations of macro-elements (K, Mg, Ca, S, and Al, mg g^{-1}) and micronutrients (Zn, Mn, Cu, Fe, B, mg kg^{-1}) in young shoots under different light intensity and P levels.

Nutrition	P level	Light Intensity			Significance		
		FL	ML	LL	light	Plevel	L×P
K	+P	23.32 ± 0.69a	20.36 ± 0.36c	15.62 ± 0.56d	$p < 0.001$	$p < 0.001$	$p < 0.001$
	-P	21.96 ± 0.52b	15.76 ± 0.44d	14.16 ± 0.68e			
S	+P	2.35 ± 0.27b	1.84 ± 0.29bc	1.63 ± 0.26c	$p < 0.001$	$p < 0.001$	$p < 0.001$
	-P	3.76 ± 0.34a	2.16 ± 0.27b	1.64 ± 0.3c			
Ca	+P	3.27 ± 0.3a	1.66 ± 0.21bc	1.69 ± 0.34bc	$p < 0.001$	$p < 0.001$	$p < 0.001$
	-P	2.08 ± 0.31b	1.71 ± 0.24b	1.17 ± 0.36c			
Al	+P	0.77 ± 0.05a	0.74 ± 0.02ab	0.68 ± 0.08bc	$p < 0.001$	$p < 0.05$	$p < 0.05$
	-P	0.8 ± 0.01a	0.69 ± 0.02b	0.62 ± 0.03c			
Mg	+P	0.91 ± 0.37b	0.96 ± 0.26b	1.98 ± 0.18a	$p < 0.001$	$p < 0.05$	$p < 0.001$
	-P	0.95 ± 0.39b	1.11 ± 0.33b	1.11 ± 0.26b			
Fe	+P	137.1 ± 2.39a	113.29 ± 2.42c	111.79 ± 2.12c	$p < 0.001$	$p < 0.001$	$p < 0.001$
	-P	131.7 ± 1.14b	114.62 ± 2.71c	86.21 ± 0.74d			
B	+P	30.56 ± 2.31a	27.22 ± 1.53b	22.76 ± 0.89c	$p < 0.001$	$p < 0.001$	ns
	-P	29.55 ± 1.2ab	23.48 ± 0.57c	21.12 ± 0.97c			
Cu	+P	29.54 ± 0.4a	19.65 ± 0.45c	19.42 ± 0.24c	$p < 0.001$	$p < 0.001$	$p < 0.001$
	-P	21.73 ± 0.86b	19.49 ± 0.29c	19.54 ± 0.19c			
Zn	+P	38.46 ± 1.12c	43.01 ± 0.35b	47.11 ± 0.6a	$p < 0.001$	$p < 0.001$	$p < 0.01$
	-P	35.2 ± 0.51d	42.06 ± 0.44b	47.03 ± 0.56a			
Mn	+P	798.59 ± 54.15a	754.57 ± 52.49a	404.8 ± 88.77c	$p < 0.001$	$p < 0.001$	$p < 0.01$
	-P	751.74 ± 82.45a	545.36 ± 60.1b	357.32 ± 67.85c			

Table S5. Concentrations of macro-elements (K, Mg, Ca, S, and Al, mg g^{-1}) and micronutrients (Zn, Mn, Cu, Fe, B, mg kg^{-1}) in leaves under different light intensity and P levels.

Nutrition	P level	Light Intensity			Significance		
		FL	ML	LL	light	Plevel	L×P
K	+P	22.49 ± 1.41a	18.97 ± 0.64c	21.05 ± 0.73abc	$p < 0.001$	$p < 0.001$	$p < 0.01$
	-P	21.45 ± 2.43ab	14.07 ± 0.88d	19.18 ± 0.98bc			
S	+P	1.41 ± 0.23b	2.49 ± 0.91a	2.29 ± 0.25ab	$p < 0.001$	ns	ns
	-P	1.58 ± 0.82ab	2.52 ± 0.21a	2.22 ± 0.36ab			
Ca	+P	4.29 ± 0.25a	1.66 ± 0.31c	4.09 ± 0.3a	$p < 0.001$	$p < 0.001$	$p < 0.001$
	-P	4.23 ± 0.32a	1.54 ± 0.4c	2.61 ± 0.23b			
Al	+P	0.41 ± 0.03a	0.2 ± 0.03c	0.36 ± 0.03a	$p < 0.001$	$p < 0.001$	ns
	-P	0.39 ± 0.03a	0.15 ± 0.04d	0.29 ± 0.03b			
Mg	+P	0.73 ± 0.03c	0.88 ± 0.03b	0.84 ± 0.03b	$p < 0.001$	$p < 0.001$	$p < 0.001$
	-P	0.88 ± 0.04b	0.86 ± 0.02b	0.98 ± 0.03a			
Fe	+P	133.9 ± 7.67a	95.75 ± 10.96c	133.52 ± 2.89a	$p < 0.001$	$p < 0.001$	$p < 0.01$
	-P	125.75 ± 13.28ab	60.75 ± 9.07d	112.44 ± 10.96bc			
B	+P	35.36 ± 0.61a	18.65 ± 0.36e	21.9 ± 0.29c	$p < 0.001$	$p < 0.001$	$p < 0.001$
	-P	24.3 ± 0.78b	14.89 ± 0.21f	20.42 ± 1.57d			
Cu	+P	21.92 ± 0.44a	14.91 ± 1.06d	20.54 ± 0.8b	$p < 0.001$	$p < 0.05$	$p < 0.05$
	-P	21.9 ± 1.08a	14.51 ± 0.21d	18.96 ± 0.24c			
Zn	+P	28.02 ± 1.43a	16.63 ± 0.57e	20.21 ± 0.33c	$p < 0.001$	$p < 0.001$	$p < 0.001$
	-P	22.67 ± 0.53b	13.59 ± 0.27f	18.33 ± 0.35d			
Mn	+P	349.22 ± 5.44e	502.4 ± 16.21a	404.04 ± 9.6c	$p < 0.001$	$p < 0.001$	ns
	-P	310.1 ± 6.11f	462.72 ± 4.87b	367.6 ± 3.08d			

Table S6. Light intensity and P interaction effect on Primary metabolites and/or related anaplerotic pathway metabolites to carbohydrates and amino acids of Longjing-43 cultivar measured by GC×GC-TOF/MS analysis (Data normalise to 1 based on treatment (FL+P).

Metabolites	Chemical Structure	Mass	1st Dim Time (Minutes)	2nd Dim Time (Seconds)	CAS	Organ	P level	Light Intensity: Full Light (FL)	Medium Light (ML)	Low Light (LL)	Significance: FL	ML	LL
1D-myo-inositol-3P	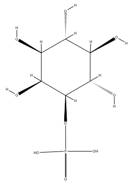	29	2.33	7.3	2831-74-5	YS	+P	1 ± 0.09b	0.55 ± 0.04d	0.79 ± 0.05c	***	***	***
							-P	1.1 ± 0.04b	2.06 ± 0.21a	0.97 ± 0.05b			
						Leaves	+P	1 ± 0.95d	1.74 ± 0.08d	3.8 ± 0.49c	***	***	***
							-P	6.95 ± 0.31b	9.42 ± 0.55a	0.92 ± 0.47d			
						Roots	+P	1 ± 0.1b	0.54 ± 0.01d	0.64 ± 0.02cd	***	***	**
							-P	1.36 ± 0.2a	0.62 ± 0.01d	0.79 ± 0.01c			
1D-myo-inositol-1P	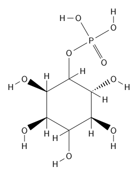	259	33.5	1.86	15421-51-9	YS	+P	1 ± 0.09b	0.52 ± 0.04d	0.76 ± 0.05c	***	***	***
							-P	1.09 ± 0.04b	2.05 ± 0.21a	0.96 ± 0.05b			
						Leaves	+P	1 ± 0.42d	2.45 ± 0.06b	1.23 ± 0.06d	***	***	***
							-P	1.81 ± 0.14c	3.4 ± 0.12a	1.17 ± 0.13d			
						Roots	+P	1 ± 0.04ab	1.1 ± 0.01a	0.97 ± 0.03b	**	***	ns
							-P	0.76 ± 0.12c	0.88 ± 0.08bc	0.81 ± 0.12c			
myo-inositol	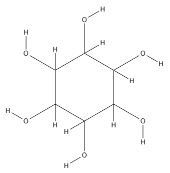	44	37.2	1.4	87-89-8	YS	+P	1 ± 0.03c	1.53 ± 0.03a	1.08 ± 0.03b	***	***	***
							-P	0.83 ± 0.01d	1.49 ± 0.1a	0.84 ± 0.01d			
						Leaves	+P	1 ± 0.64c	0.95 ± 0.06c	1.48 ± 0.07ab	**	***	ns
							-P	1.52 ± 0.04a	1.74 ± 0.05a	1.04 ± 0.15bc			
						Roots	+P	1 ± 0.12b	1.21 ± 0.02a	1.02 ± 0.06b	***	***	ns
							-P	1.17 ± 0.18ab	1.26 ± 0.01a	0.68 ± 0.12c			
D-Glucuronate	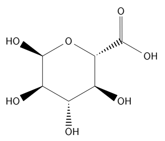	363	35.6	2.05	6556-12-3	YS	+P	1 ± 0.09b	0.49 ± 0.04d	0.75 ± 0.05c	***	***	***
							-P	1.08 ± 0.05b	2.05 ± 0.21a	0.95 ± 0.05b			
						Leaves	+P	1 ± 0.64e	0.95 ± 0.06e	4.45 ± 0.31b	***	***	***
							-P	3.25 ± 0.2c	1.74 ± 0.05d	6.8 ± 0.2a			
						Roots	+P	1 ± 0.26a	0.96 ± 0.01a	0.81 ± 0.05a	***	**	**
							-P	0.79 ± 0.09a	1 ± 0.01a	0.54 ± 0.09b			

Continue on the next page

Light intensity and P interaction effect on Primary metabolites and/or related anaplerotic pathway metabolites to carbohydrates and amino acids of Longjing-43 cultivar measured by GC×GC-TOF/MS analysis (Data normalise to 1 based on treatment (FL+P) (*cont.*).

Metabolites	Chemical Structure	Mass	1st Dim Time (Minutes)	2nd Dim Time (Seconds)	CAS	Organ	P level	Light Intensity: Full Light (FL)	Medium Light (ML)	Low Light (LL)	Significance: FL	ML	LL
D-Fructose	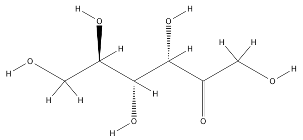	204	21.5	2.26	57-48-7	YS	+P	1 ± 0.03d	1.63 ± 0.03a	1.11 ± 0.03c	***	***	*
							-P	0.79 ± 0.01e	1.42 ± 0.09b	0.82 ± 0.01e			
						Leaves	+P	1 ± 0.64b	0.95 ± 0.06b	1.72 ± 0.04a	*	***	***
							-P	1.65 ± 0.05a	1.74 ± 0.05a	1.49 ± 0.09a			
						Roots	+P	1 ± 0.09bc	0.72 ± 0.05d	0.85 ± 0.05cd	***	***	***
							-P	1.33 ± 0.11a	1.12 ± 0.22b	0.83 ± 0cd			
D-Mannose	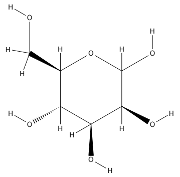	103	29.5	2.5	31103-86-3	YS	+P	1 ± 0.01e	2.05 ± 0.15b	1.64 ± 0.06c	***	***	ns
							-P	1.4 ± 0.04d	2.44 ± 0.04a	0.97 ± 0.01e			
						Leaves	+P	1 ± 0.42d	3.12 ± 0.05d	34.8 ± 4.15b	***	***	***
							-P	21.89 ± 2c	4.92 ± 0.38d	66.59 ± 4.03a			
						Roots	+P	1 ± 0.28bc	0.75 ± 0.24c	1.31 ± 0.36bc	***	***	***
							-P	0.78 ± 0.04c	2.85 ± 0.37a	1.86 ± 1.03b			
D-Mannose-6P	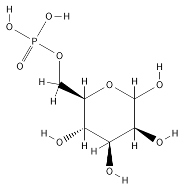	260	28.5	2.69	3672-15-9	YS	+P	1 ± 0.07bc	0.84 ± 0.04d	0.87 ± 0.04d	***	***	***
							-P	1.04 ± 0.04b	1.88 ± 0.13a	0.9 ± 0.05cd			
						Leaves	+P	1 ± 0.11d	1.3 ± 0.21d	17.62 ± 2.05b	***	***	***
							-P	10.63 ± 1.11c	0.78 ± 0.07d	33.14 ± 1.95a			
						Roots	+P	1 ± 0.13a	0.99 ± 0.01a	0.8 ± 0.08a	***	**	*
							-P	0.93 ± 0.13a	1.01 ± 0a	0.49 ± 0.25b			
D-Gluconate	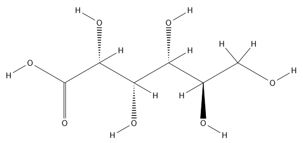	174	35	2.1	526-95-4	YS	+P	1 ± 0.02d	1.71 ± 0.04a	1.13 ± 0.03c	***	***	**
							-P	0.76 ± 0.02e	1.36 ± 0.08b	0.8 ± 0.01e			
						Leaves	+P	1 ± 0.11b	1.3 ± 0.21a	1.06 ± 0.04b	**	***	***
							-P	1.06 ± 0.03b	0.78 ± 0.07c	0.74 ± 0.06c			
						Roots	+P	1 ± 0.13a	0.99 ± 0.01a	0.64 ± 0.09b	***	***	***
							-P	0.93 ± 0.13a	1.01 ± 0a	0.09 ± 0.01c			

Continue on the next page

Light intensity and P interaction effect on Primary metabolites and/or related anaplerotic pathway metabolites to carbohydrates and amino acids of Longjing-43 cultivar measured by GC×GC-TOF/MS analysis (Data normalise to 1 based on treatment (FL+P) (*cont.*).

Metabolites	Chemical Structure	Mass	1st Dim Time (Minutes)	2nd Dim Time (Seconds)	CAS	Organ	P level	Light Intensity			Significance		
								Full Light (FL)	Medium Light (ML)	Low Light (LL)	FL	ML	LL
D-gluconate 6P	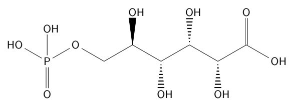	361	36.1	2.35	921-62-0	YS	+P	1 ± 0.06bc	1.08 ± 0.04b	0.95 ± 0.04cd	***	***	***
							-P	1.02 ± 0.03bc	1.77 ± 0.08a	0.87 ± 0.05d			
						Leaves	+P	1 ± 0.55c	0.96 ± 0.21c	2.9 ± 0.35b	***	***	***
							-P	4.02 ± 0.17a	3.05 ± 0.19b	0.91 ± 0.39c			
						Roots	+P	1 ± 0.08bc	1.14 ± 0.24b	0.98 ± 0.09bc	***	***	***
							-P	0.82 ± 0c	2.18 ± 0.33a	0.86 ± 0.01bc			
D-Ribulose5P	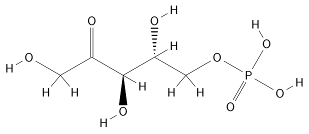	243	19.2	2.56	551-85-9	YS	+P	1 ± 0.02d	1.73 ± 0.03a	1.16 ± 0.04c	***	***	***
							-P	0.74 ± 0.01e	1.42 ± 0.02b	0.74 ± 0e			
						Leaves	+P	1 ± 0.76bc	0.78 ± 0.15c	1.59 ± 0.08a	***	ns	ns
							-P	1.22 ± 0.05abc	1.1 ± 0.1abc	1.42 ± 0.08ab			
						Roots	+P	1 ± 0.13b	0.5 ± 0.02d	0.64 ± 0.02c	***	***	***
							-P	1.31 ± 0.07a	0.97 ± 0.08b	0.72 ± 0.03c			
D-Ribose-5P	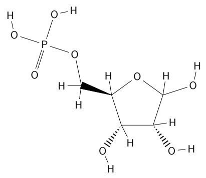	39	10.7	1.96	4300-28-1	YS	+P	1 ± 0.05c	1.32 ± 0.04b	1.03 ± 0.04c	***	*	***
							-P	0.99 ± 0.03c	1.64 ± 0.04a	0.82 ± 0.05d			
						Leaves	+P	1 ± 0.39e	0.27 ± 0f	4.94 ± 0.56b	***	***	***
							-P	3.96 ± 0.24c	2.13 ± 0.27d	8.78 ± 0.46a			
						Roots	+P	1 ± 0.16b	0.76 ± 0.01b	0.78 ± 0.02b	***	***	***
							-P	1.56 ± 0.29a	1 ± 0.07b	0.76 ± 0.08b			
Ribose	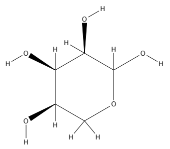	50	7.2	1.41	50-69-1	YS	+P	1 ± 0.04c	1.73 ± 0.02a	0.73 ± 0.06d	***	***	***
							-P	1.07 ± 0.05c	1.76 ± 0.03a	1.21 ± 0.03b			
						Leaves	+P	1 ± 0.08e	0.61 ± 0.13f	2.85 ± 0.2b	***	***	ns
							-P	2.54 ± 0.12c	2.05 ± 0.11d	4.11 ± 0.23a			
						Roots	+P	1 ± 0.21d	5.09 ± 0.03b	5.26 ± 0.52b	***	***	***
							-P	3.75 ± 1.07c	7.98 ± 1.29a	4.27 ± 0.02bc			

Continue on the next page

Light intensity and P interaction effect on Primary metabolites and/or related anaplerotic pathway metabolites to carbohydrates and amino acids of Longjing-43 cultivar measured by GC×GC-TOF/MS analysis (Data normalise to 1 based on treatment (FL+P) (*cont.*).

Metabolites	Chemical Structure	Mass	1st Dim Time (Minutes)	2nd Dim Time (Seconds)	CAS	Organ	P level	Light Intensity: Full Light (FL)	Light Intensity: Medium Light (ML)	Light Intensity: Low Light (LL)	Significance: FL	Significance: ML	Significance: LL
D-Xylulose-5P	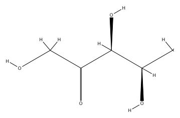	217	16.1	2.08	60802-29-1	YS	+P	1 ± 0.09d	2.43 ± 0.04a	1.64 ± 0.04b	***	***	***
							-P	1.07 ± 0.03d	1.53 ± 0.07c	0.81 ± 0.06e			
						Leaves	+P	1 ± 0.77c	1.99 ± 0.1a	1.28 ± 0.15bc	***	***	ns
							-P	1.77 ± 0.03ab	2.11 ± 0.02a	0.03 ± 0.01d			
						Roots	+P	1 ± 0.06d	0.6 ± 0.02e	1.81 ± 0.05bc	***	***	***
							-P	1.62 ± 0.15c	2.07 ± 0.37b	3.05 ± 0.14a			
D-Xylulose	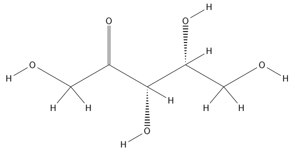	69	10.7	1.86	551-84-8	YS	+P	1 ± 0.09c	2.37 ± 0.04a	1.6 ± 0.04b	***	***	***
							-P	1.07 ± 0.03c	1.51 ± 0.07b	0.84 ± 0.06d			
						Leaves	+P	1 ± 0.4d	1.55 ± 0.47d	10.12 ± 1.09b	***	***	***
							-P	6.2 ± 0.59c	0.64 ± 0.08d	18.76 ± 1.11a			
						Roots	+P	1 ± 0.06d	0.6 ± 0.02e	1.81 ± 0.05bc	***	***	***
							-P	1.62 ± 0.15c	2.07 ± 0.37b	3.05 ± 0.14a			
D-Xylose	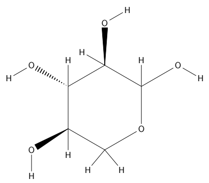	204	21.5	0.04	58-86-6	YS	+P	1 ± 0.09c	1.5 ± 0.06b	0.87 ± 0.06d	***	***	***
							-P	1.06 ± 0.02c	2.31 ± 0.04a	1.56 ± 0.04b			
						Leaves	+P	1 ± 0.1a	0.3 ± 0.01e	0.85 ± 0.02b	***	**	***
							-P	0.74 ± 0.03c	0.54 ± 0.02d	0.73 ± 0.01c			
						Roots	+P	1 ± 0.11c	0.51 ± 0.01d	0.92 ± 0.2c	***	***	***
							-P	1.35 ± 0.02b	0.39 ± 0.03d	1.72 ± 0.13a			
Xylitol	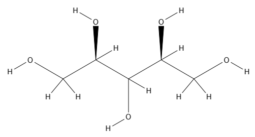	44	18.7	1.5	87-99-0	YS	+P	1 ± 0.08cd	2.14 ± 0.03a	1.47 ± 0.04b	***	***	***
							-P	1.07 ± 0.02c	1.43 ± 0.06b	0.94 ± 0.06d			
						Leaves	+P	1 ± 0.1d	0.3 ± 0.01d	8.4 ± 0.93b	***	***	***
							-P	5.15 ± 0.52c	0.54 ± 0.02d	15.38 ± 0.87a			
						Roots	+P	1 ± 0.05b	1.42 ± 0.05a	1.02 ± 0.06b	***	***	***
							-P	0.63 ± 0.02c	0.6 ± 0.3c	0.54 ± 0.1c			

Continue on the next page

Light intensity and P interaction effect on Primary metabolites and/or related anaplerotic pathway metabolites to carbohydrates and amino acids of Longjing-43 cultivar measured by GC×GC-TOF/MS analysis (Data normalise to 1 based on treatment (FL+P) (*cont.*).

Metabolites	Chemical Structure	Mass	1st Dim Time (Minutes)	2nd Dim Time (Seconds)	CAS	Organ	P level	Light Intensity: Full Light (FL)	Light Intensity: Medium Light (ML)	Light Intensity: Low Light (LL)	Significance: FL	Significance: ML	Significance: LL
L-Xylulose	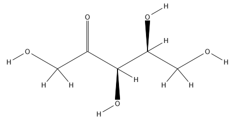	217	16.1	2.08	527-50-4	YS	+P	1 ± 0.09cd	2.18 ± 0.03a	1.49 ± 0.04b	***	***	***
							-P	1.06 ± 0.02c	1.45 ± 0.06b	0.93 ± 0.06d			
						Leaves	+P	1 ± 0.28b	0.71 ± 0.02c	1.57 ± 0.11a	***	***	ns
							-P	1.69 ± 0.1a	0.87 ± 0.02bc	0.85 ± 0.15bc			
						Roots	+P	1 ± 0.15b	0.47 ± 0.01cd	0.53 ± 0.1cd	***	***	***
							-P	1.49 ± 0.23a	0.42 ± 0d	0.7 ± 0.22c			
L-Arabitol	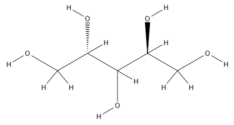	217	18.8	2.16	7643-75-6	YS	+P	1 ± 0.09cd	2.21 ± 0.03a	1.5 ± 0.04b	***	***	***
							-P	1.05 ± 0.02c	1.47 ± 0.06b	0.91 ± 0.06d			
						Leaves	+P	1 ± 0.01a	0.51 ± 0.02e	0.88 ± 0.04b	***	***	***
							-P	0.74 ± 0.04c	0.35 ± 0.03f	0.58 ± 0.06d			
						Roots	+P	1 ± 0.22ab	0.94 ± 0.3ab	0.75 ± 0.18ab	**	ns	ns
							-P	1.11 ± 0.11ab	1.18 ± 0.26a	0.7 ± 0.36b			
L-Arabinose	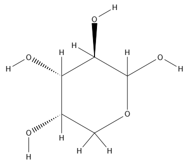	89	11.7	2.3	147-81-9	YS	+P	1 ± 0.05d	1.84 ± 0.03a	1.21 ± 0.03c	***	***	***
							-P	0.9 ± 0.04e	1.57 ± 0.06b	0.63 ± 0.06f			
						Leaves	+P	1 ± 0.34a	0.27 ± 0.01b	0.97 ± 0.08a	***	***	***
							-P	0.76 ± 0.03a	0.49 ± 0.02b	0.37 ± 0.05b			
						Roots	+P	1 ± 1.36c	0.14 ± 0.02c	2.87 ± 0.09b	***	***	***
							-P	0.24 ± 0c	6.77 ± 1.56a	5.66 ± 0.18a			
L-Serine	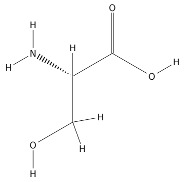	116	10.7	2.05	56-45-1	YS	+P	1 ± 0.03d	2.23 ± 0.04a	1.45 ± 0.04b	***	***	***
							-P	0.95 ± 0.06d	1.26 ± 0.06c	0.73 ± 0.06e			
						Leaves	+P	1 ± 0.64e	3.7 ± 0.02d	11.22 ± 1.12b	***	***	***
							-P	7.3 ± 0.71c	0.38 ± 0.03e	19.52 ± 1.17a			
						Roots	+P	1 ± 0.08a	0.52 ± 0.1c	0.54 ± 0.09c	***	***	ns
							-P	0.74 ± 0.12b	0.99 ± 0.12a	0.48 ± 0.12c			

Continue on the next page

Light intensity and P interaction effect on Primary metabolites and/or related anaplerotic pathway metabolites to carbohydrates and amino acids of Longjing-43 cultivar measured by GC×GC-TOF/MS analysis (Data normalise to 1 based on treatment (FL+P) (*cont.*).

Metabolites	Chemical Structure	Mass	1st Dim Time (Minutes)	2nd Dim Time (Seconds)	CAS	Organ	P level	Light Intensity: Full Light (FL)	Light Intensity: Medium Light (ML)	Light Intensity: Low Light (LL)	Significance: FL	Significance: ML	Significance: LL
Glycine	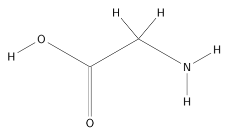	178	36.8	0.04	56-40-6	YS	+P	1 ± 0.05d	1.81 ± 0.02a	1.19 ± 0.03c	***	***	***
							-P	0.9 ± 0.03e	1.51 ± 0.06b	0.65 ± 0.05f			
						Leaves	+P	1 ± 0.26d	0.32 ± 0.01d	23.63 ± 2.87b	***	***	***
							-P	13.94 ± 1.53c	0.26 ± 0.01d	45.4 ± 2.76a			
						Roots	+P	1 ± 0.19cd	0.78 ± 0.17d	1.14 ± 0.14bcd	**	***	ns
							-P	1.37 ± 0.28abc	1.45 ± 0.02ab	1.72 ± 0.33a			
Threonine	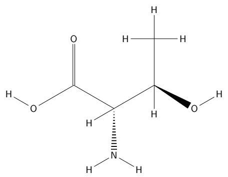	102	16.2	2.2	72-19-5	YS	+P	1 ± 0.03d	2.18 ± 0.04a	1.42 ± 0.04b	***	***	***
							-P	0.95 ± 0.06d	1.24 ± 0.06c	0.75 ± 0.06e			
						Leaves	+P	1 ± 0.17c	0.95 ± 0.01c	0.6 ± 0.02c	***	***	***
							-P	2.9 ± 0.48b	8.32 ± 0.47a	0.55 ± 0.01c			
						Roots	+P	1 ± 0.08a	0.33 ± 0c	0.46 ± 0.09c	***	ns	***
							-P	0.65 ± 0.07b	0.96 ± 0.22a	0.4 ± 0c			
L-Homoserine	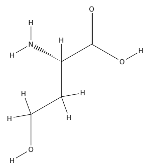	74	10.5	1.62	56-45-1	YS	+P	1 ± 0.05d	1.79 ± 0.02a	1.19 ± 0.03c	***	***	***
							-P	0.9 ± 0.03e	1.49 ± 0.07b	0.66 ± 0.05f			
						Leaves	+P	1 ± 0.1e	1.12 ± 0.01e	1.6 ± 0.09d	***	***	***
							-P	1.97 ± 0.08c	2.71 ± 0.1a	2.18 ± 0.03b			
						Roots	+P	1 ± 0.13b	0.52 ± 0c	0.81 ± 0.11bc	***	***	***
							-P	1.55 ± 0.12a	1.48 ± 0.34a	0.87 ± 0.14b			
O-Phospho-L-homoserine	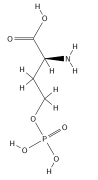	68	18.4	2.24	4210-66-6	YS	+P	1 ± 0.06d	2.31 ± 0.05a	1.49 ± 0.04b	***	***	***
							-P	1.04 ± 0.04d	1.27 ± 0.07c	0.76 ± 0.07e			
						Leaves	+P	1 ± 0.31d	1.64 ± 0.05d	9.93 ± 1.04b	***	***	***
							-P	6.62 ± 0.56c	1.58 ± 0.02d	17.64 ± 1.02a			
						Roots	+P	1 ± 0.08ab	0.89 ± 0.02b	0.97 ± 0ab	***	ns	ns
							-P	1.09 ± 0.15a	1.09 ± 0.04a	1.03 ± 0.02a			

Continue on the next page

Light intensity and P interaction effect on Primary metabolites and/or related anaplerotic pathway metabolites to carbohydrates and amino acids of Longjing-43 cultivar measured by GC×GC-TOF/MS analysis (Data normalise to 1 based on treatment (FL+P) (*cont.*).

Metabolites	Chemical Structure	Mass	1st Dim Time (Minutes)	2nd Dim Time (Seconds)	CAS	Organ	P level	Light Intensity: Full Light (FL)	Light Intensity: Medium Light (ML)	Light Intensity: Low Light (LL)	Significance: FL	Significance: ML	Significance: LL
L-Leucine	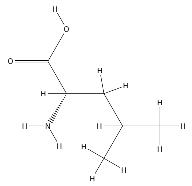	200	17.3	2.84	61-90-5	YS	+P	1 ± 0.05d	1.74 ± 0.02a	1.17 ± 0.04c	***	***	***
							-P	0.93 ± 0.03d	1.5 ± 0.07b	0.71 ± 0.05e			
						Leaves	+P	1 ± 0.04d	1.04 ± 0.01d	2.02 ± 0.13cd	***	***	***
							-P	7.45 ± 1.18b	20.79 ± 1.24a	3.07 ± 0.15c			
						Roots	+P	1 ± 0.07b	0.63 ± 0.04d	0.51 ± 0.03e	***	***	***
							-P	1.27 ± 0.01a	0.9 ± 0.07c	0.34 ± 0.02f			
L-IsoLeucine	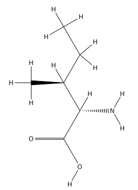	158	11.2	1.9	73-32-5	YS	+P	1 ± 0.06d	1.27 ± 0.06c	0.78 ± 0.07e	***	***	***
							-P	1.05 ± 0.04d	2.28 ± 0.04a	1.47 ± 0.04b			
						Leaves	+P	1 ± 0.27e	1.59 ± 0.05d	4.75 ± 0.4b	***	***	***
							-P	3.41 ± 0.25c	0.9 ± 0.11e	7.62 ± 0.41a			
						Roots	+P	1 ± 0.07b	0.63 ± 0.04d	0.51 ± 0.03e	***	***	***
							-P	1.27 ± 0.01a	0.9 ± 0.07c	0.34 ± 0.02f			
L-Valine	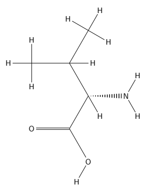	142	16.4	2.86	72-18-4	YS	+P	1 ± 0.04d	1.65 ± 0.02a	1.08 ± 0.03c	***	***	***
							-P	0.88 ± 0.03e	1.26 ± 0.04b	0.65 ± 0.05f			
						Leaves	+P	1 ± 0.18e	1.24 ± 0.24e	5.88 ± 0.56b	***	***	***
							-P	4.7 ± 0.23c	2.62 ± 0.28d	9.88 ± 0.5a			
						Roots	+P	1 ± 0a	0.49 ± 0.03d	0.4 ± 0.02e	***	***	***
							-P	0.79 ± 0.05b	0.7 ± 0.06c	0.27 ± 0.02f			
Phenylpyruvate	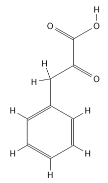	120	15.9	2.97	156-06-9	YS	+P	1 ± 0.04c	1.62 ± 0.02a	1.06 ± 0.03c	***	***	***
							-P	0.88 ± 0.03d	1.25 ± 0.04b	0.65 ± 0.05e			
						Leaves	+P	1 ± 0.02d	0.55 ± 0.01e	3.02 ± 0.24b	***	***	***
							-P	2.51 ± 0.21c	0.68 ± 0.01e	3.89 ± 0.16a			
						Roots	+P	1 ± 0.17c	1.48 ± 0.06a	1.16 ± 0.01b	***	***	***
							-P	0.66 ± 0.07d	0.85 ± 0.08c	0.87 ± 0.04c			

Continue on the next page

Light intensity and P interaction effect on Primary metabolites and/or related anaplerotic pathway metabolites to carbohydrates and amino acids of Longjing-43 cultivar measured by GC×GC-TOF/MS analysis (Data normalise to 1 based on treatment (FL+P) (*cont.*).

Metabolites	Chemical Structure	Mass	1st Dim Time (Minutes)	2nd Dim Time (Seconds)	CAS	Organ	P level	Light Intensity: Full Light (FL)	Light Intensity: Medium Light (ML)	Light Intensity: Low Light (LL)	Significance: FL	Significance: ML	Significance: LL
L-Phenylalanine	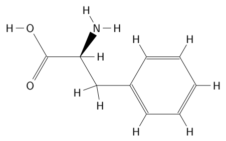	154	20.7	3.34	63-91-2	YS	+P	1 ± 0.03d	1.6 ± 0.08b	0.78 ± 0.06e	***	***	***
							-P	1.07 ± 0.06d	1.85 ± 0.03a	1.25 ± 0.04c			
						Leaves	+P	1 ± 0.42b	0.43 ± 0c	0.98 ± 0.07b	***	***	**
							-P	0.96 ± 0.03b	0.87 ± 0.09b	1.49 ± 0.08a			
						Roots	+P	1 ± 0.07b	1.25 ± 0.05a	0.96 ± 0.05b	***	***	***
							-P	1.23 ± 0.18a	0.92 ± 0b	0.64 ± 0.07c			
4-Hydroxy-phenylpyruvate	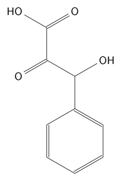	133	16	1.44	156-39-8	YS	+P	1 ± 0.04c	1.59 ± 0.02a	1.04 ± 0.03c	***	***	***
							-P	0.88 ± 0.03d	1.25 ± 0.05b	0.65 ± 0.05e			
						Leaves	+P	1 ± 0.43b	0.66 ± 0.01c	1.49 ± 0.11a	***	ns	***
							-P	1.52 ± 0.1a	0.68 ± 0bc	0.58 ± 0.06c			
						Roots	+P	1 ± 0.14b	1.1 ± 0.12b	1.31 ± 0.11b	***	***	***
							-P	1.07 ± 0.08b	3.03 ± 0.61a	1.22 ± 0.21b			
L-Tyrosine	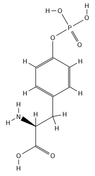	218	24.7	2.99	60-18-4	YS	+P	1 ± 0.05d	1.71 ± 0.03a	1.16 ± 0.04c	***	***	***
							-P	0.93 ± 0.03d	1.48 ± 0.08b	0.74 ± 0.05e			
						Leaves	+P	1 ± 0.06d	0.85 ± 0.43d	2.04 ± 0.11c	*	***	***
							-P	2.87 ± 0.15b	3.61 ± 0.53a	2.5 ± 0.01bc			
						Roots	+P	1 ± 0.19b	1.21 ± 0.01a	1.11 ± 0.01ab	**	*	ns
							-P	1.03 ± 0.1b	1.08 ± 0.05ab	0.98 ± 0.01b			
L-Tyroptophan	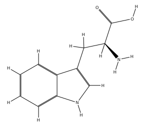	146	17	2.65	73-22-3	YS	+P	1 ± 0.02c	1.61 ± 0.07b	0.85 ± 0.05d	***	***	***
							-P	1.06 ± 0.1c	2.49 ± 0.03a	1.57 ± 0.05b			
						Leaves	+P	1 ± 0.19de	0.51 ± 0.2e	6.25 ± 0.69b	***	***	***
							-P	4.18 ± 0.34c	1.45 ± 0.08d	11.47 ± 0.58a			
						Roots	+P	1 ± 0.19b	1.21 ± 0.01a	1.11 ± 0.01ab	**	*	ns
							-P	1.03 ± 0.1b	1.08 ± 0.05ab	0.98 ± 0.01b			

Continue on the next page

Light intensity and P interaction effect on Primary metabolites and/or related anaplerotic pathway metabolites to carbohydrates and amino acids of Longjing-43 cultivar measured by GC×GC-TOF/MS analysis (Data normalise to 1 based on treatment (FL+P) (*cont.*).

Metabolites	Chemical Structure	Mass	1st Dim Time (Minutes)	2nd Dim Time (Seconds)	CAS	Organ	P level	Light Intensity: Full Light (FL)	Light Intensity: Medium Light (ML)	Light Intensity: Low Light (LL)	Significance: FL	Significance: ML	Significance: LL
Oxalic acid	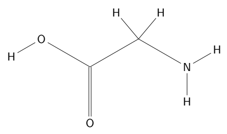	57	18.4	2.3	144-62-7	YS	+P	1 ± 0.08c	2.29 ± 0.04a	1.44 ± 0.05b	***	***	***
							-P	0.91 ± 0.02d	1.45 ± 0.06b	0.74 ± 0.05e			
						Leaves	+P	1 ± 0.39b	0.17 ± 0.07c	0.28 ± 0.01c	***	***	***
							-P	0.81 ± 0.1b	1.85 ± 0.13a	0.16 ± 0.03c			
						Roots	+P	1 ± 0.18c	1.2 ± 0.01b	1.01 ± 0.07c	***	*	***
							-P	0.93 ± 0.12c	1.38 ± 0.03a	0.66 ± 0.04d			
Citrate	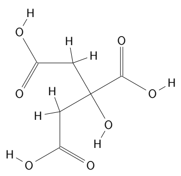	101	9.5	2.21	77-92-9	YS	+P	1 ± 0.09c	2.35 ± 0.03a	1.48 ± 0.05b	***	***	***
							-P	0.94 ± 0.02c	1.5 ± 0.06b	0.79 ± 0.05d			
						Leaves	+P	1 ± 0.42a	0.4 ± 0.12c	0.61 ± 0.07bc	***	**	ns
							-P	0.87 ± 0.04ab	0.7 ± 0.01abc	0.45 ± 0.12c			
						Roots	+P	1 ± 0.27bc	0.96 ± 0.18bc	1.18 ± 0.46b	***	***	***
							-P	0.59 ± 0.33bc	4.76 ± 0.83a	0.33 ± 0.04c			
Isocitrate	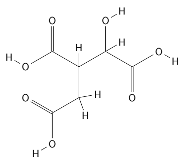	73	22.5	2.4	320-77-4	YS	+P	1 ± 0.09c	2.32 ± 0.03a	1.47 ± 0.05b	***	***	***
							-P	0.94 ± 0.02c	1.5 ± 0.06b	0.79 ± 0.05d			
						Leaves	+P	1 ± 0.22a	1.02 ± 0.1a	0.95 ± 0.06ab	***	***	***
							-P	1.03 ± 0.06a	0.32 ± 0.07c	0.77 ± 0.01b			
						Roots	+P	1 ± 0.07b	0.66 ± 0.24b	3.22 ± 2.05b	***	***	***
							-P	1.23 ± 0.01b	34.03 ± 7.18a	1.37 ± 0.01b			
Oxoglutarate	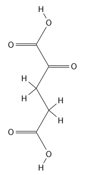	362	21.9	2.53	328-50-7	YS	+P	1 ± 0.02c	1.6 ± 0.07b	0.85 ± 0.05d	***	***	***
							-P	1.06 ± 0.1c	2.46 ± 0.03a	1.56 ± 0.05b			
						Leaves	+P	1 ± 0.21d	0.92 ± 0.03de	3.6 ± 0.36b	***	***	***
							-P	2.37 ± 0.19c	0.57 ± 0.01e	6.39 ± 0.36a			
						Roots	+P	1 ± 0.15b	0.97 ± 0b	1.03 ± 0.08b	***	***	***
							-P	1.05 ± 0.18b	1.71 ± 0.17a	0.91 ± 0.01b			

Continue on the next page

Light intensity and P interaction effect on Primary metabolites and/or related anaplerotic pathway metabolites to carbohydrates and amino acids of Longjing-43 cultivar measured by GC×GC-TOF/MS analysis (Data normalise to 1 based on treatment (FL+P) (*cont.*).

Metabolites	Chemical Structure	Mass	1st Dim Time (Minutes)	2nd Dim Time (Seconds)	CAS	Organ	P level	Light Intensity: Full Light (FL)	Light Intensity: Medium Light (ML)	Light Intensity: Low Light (LL)	Significance: FL	Significance: ML	Significance: LL
Succinate	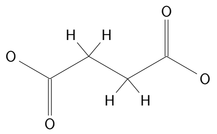	147	27.3	2.65	110-15-6	YS	+P	1 ± 0.08c	2.27 ± 0.03a	1.44 ± 0.05b	***	***	***
							-P	0.92 ± 0.02c	1.47 ± 0.06b	0.77 ± 0.05d			
						Leaves	+P	1 ± 0.14d	0.66 ± 0.04de	3.24 ± 0.3b	***	***	***
							-P	2.18 ± 0.2c	0.31 ± 0.03e	4.51 ± 0.41a			
						Roots	+P	1 ± 0.21b	1.57 ± 0.18a	0.94 ± 0.07b	***	***	***
							-P	0.58 ± 0.03c	0.48 ± 0.02c	0.4 ± 0.03c			
Fumarate	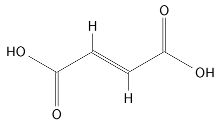	298	22.9	3.5	110-17-8	YS	+P	1 ± 0.06c	2.05 ± 0.03a	1.33 ± 0.03b	***	***	***
							-P	1 ± 0.04c	1.33 ± 0.06b	0.75 ± 0.06d			
						Leaves	+P	1 ± 0.81bc	3.11 ± 0.22a	0.67 ± 0.2cd	***	***	***
							-P	0.97 ± 0.17c	1.62 ± 0.21b	0.09 ± 0d			
						Roots	+P	1 ± 0.02e	1.17 ± 0.08d	1.31 ± 0.04c	***	***	***
							-P	0.66 ± 0.09f	1.64 ± 0.01a	1.5 ± 0.07b			
Malate	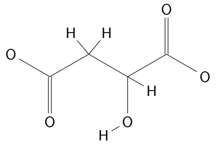	77	6	0.1	97-67-6	YS	+P	1 ± 0.06c	2.05 ± 0.03a	1.33 ± 0.04b	***	***	***
							-P	0.99 ± 0.03c	1.33 ± 0.06b	0.75 ± 0.06d			
						Leaves	+P	1 ± 0.94a	0.11 ± 0.03b	1.17 ± 0.17a	**	*	***
							-P	0.73 ± 0.04ab	0.49 ± 0.02ab	0.05 ± 0.01b			
						Roots	+P	1 ± 0.11b	0.91 ± 0bc	0.93 ± 0.04bc	***	***	***
							-P	1.17 ± 0.06a	1.19 ± 0.05a	0.85 ± 0.01c			
L-Asparate	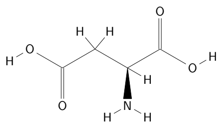	89	19.4	2.82	56-84-8	YS	+P	1 ± 0.09c	2.29 ± 0.03a	1.46 ± 0.05b	***	***	***
							-P	0.95 ± 0.02c	1.5 ± 0.07b	0.8 ± 0.05d			
						Leaves	+P	1 ± 0.06d	1.56 ± 0.11b	1.41 ± 0.07c	***	***	***
							-P	1.29 ± 0.04c	1.1 ± 0.1d	2.03 ± 0.09a			
						Roots	+P	1 ± 0.07c	1.31 ± 0.23c	10.92 ± 1.84b	***	***	***
							-P	0.8 ± 0c	0.51 ± 0.11c	24.67 ± 0.78a			

Continue on the next page

Light intensity and P interaction effect on Primary metabolites and/or related anaplerotic pathway metabolites to carbohydrates and amino acids of Longjing-43 cultivar measured by GC×GC-TOF/MS analysis (Data normalise to 1 based on treatment (FL+P) (*cont.*).

Metabolites	Chemical Structure	Mass	1st Dim Time (Minutes)	2nd Dim Time (Seconds)	CAS	Organ	P level	Light Intensity			Significance		
								Full Light (FL)	Medium Light (ML)	Low Light (LL)	FL	ML	LL
L-Alanine	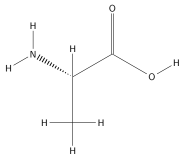	44	31.7	1.44	56-41-7	YS	+P	1 ± 0.03c	1.32 ± 0.06b	0.75 ± 0.06d	***	***	***
							-P	1.02 ± 0.05c	1.96 ± 0.02a	1.29 ± 0.03b			
						Leaves	+P	1 ± 0.03a	0.35 ± 0.02e	0.95 ± 0.03ab	***	***	ns
							-P	0.92 ± 0.04b	0.73 ± 0.02c	0.66 ± 0.03d			
						Roots	+P	1 ± 0.29bc	0.25 ± 0c	0.83 ± 0.32bc	***	***	*
							-P	2.73 ± 0.56a	1.36 ± 0.26b	1.23 ± 1.17bc			
L-Asparagine	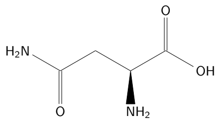	99	18.1	2.7	70-47-3	YS	+P	1 ± 0.05c	1.91 ± 0.02a	1.25 ± 0.03b	***	***	***
							-P	0.98 ± 0.03c	1.29 ± 0.06b	0.74 ± 0.06d			
						Leaves	+P	1 ± 0.12ab	1.1 ± 0.38a	0.76 ± 0.06bc	**	***	ns
							-P	0.66 ± 0.03cd	0.59 ± 0.19cd	0.39 ± 0.1d			
						Roots	+P	1 ± 0.11a	0.1 ± 0c	0.11 ± 0c	***	***	***
							-P	0.52 ± 0.11b	0.13 ± 0.01c	0.11 ± 0.02c			
L-Glutamate	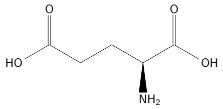	152	7.6	2.34	56-86-0	YS	+P	1 ± 0.03c	1.32 ± 0.07b	0.76 ± 0.06d	***	***	***
							-P	1.03 ± 0.05c	1.92 ± 0.02a	1.26 ± 0.04b			
						Leaves	+P	1 ± 0.23d	0.73 ± 0.04de	3.64 ± 0.36b	***	***	***
							-P	2.43 ± 0.23c	0.28 ± 0.03e	5.24 ± 0.49a			
						Roots	+P	1 ± 0.12a	0.39 ± 0.03c	0.38 ± 0.05c	***	***	***
							-P	0.65 ± 0.07b	0.22 ± 0d	0.45 ± 0.15c			
L-Glutamine	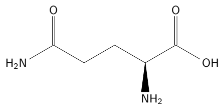	127	18.1	3.24	56-85-9	YS	+P	1 ± 0.05c	1.8 ± 0.02a	1.2 ± 0.03b	***	***	***
							-P	0.96 ± 0.03c	1.26 ± 0.06b	0.73 ± 0.06d			
						Leaves	+P	1 ± 0.05a	0.56 ± 0.03d	0.93 ± 0.03b	***	***	**
							-P	0.74 ± 0.05c	0.22 ± 0.03e	0.68 ± 0.03c			
						Roots	+P	1 ± 0.25ab	1.72 ± 0.63a	1.28 ± 0.22a	***	*	ns
							-P	0.26 ± 0b	1.28 ± 0.74a	1.4 ± 0.01a			

Continue on the next page

Light intensity and P interaction effect on Primary metabolites and/or related anaplerotic pathway metabolites to carbohydrates and amino acids of Longjing-43 cultivar measured by GC×GC-TOF/MS analysis (Data normalise to 1 based on treatment (FL+P) (*cont.*).

Metabolites	Chemical Structure	Mass	1st Dim Time (Minutes)	2nd Dim Time (Seconds)	CAS	Organ	P level	Light Intensity: Full Light (FL)	Medium Light (ML)	Low Light (LL)	Significance: FL	ML	LL
L-Proline	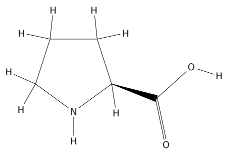	116	35	2.46	147-85-3	YS	+P	1 ± 0.11d	1.55 ± 0.08b	0.87 ± 0.06e	***	***	***
							-P	1 ± 0.02d	2.13 ± 0.03a	1.39 ± 0.05c			
						Leaves	+P	1 ± 0.39d	0.19 ± 0.01e	3.43 ± 0.38b	***	***	***
							-P	2.18 ± 0.22c	0.25 ± 0e	6.13 ± 0.29a			
						Roots	+P	1 ± 0.03a	0.7 ± 0.03c	0.71 ± 0.03c	***	***	ns
							-P	0.89 ± 0.05b	0.79 ± 0c	0.74 ± 0.12c			
Hydroxyproline	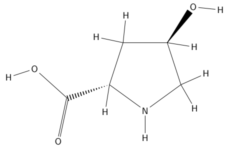	230	6.3	0.05	51-35-4	YS	+P	1 ± 0.02d	2.07 ± 0.03a	1.36 ± 0.05c	***	***	***
							-P	1 ± 0.1d	1.52 ± 0.08b	0.87 ± 0.06e			
						Leaves	+P	1 ± 0.06b	1.42 ± 0.01a	0.44 ± 0.08d	***	***	***
							-P	0.76 ± 0.08c	1.51 ± 0.02a	0.21 ± 0.01e			
						Roots	+P	1 ± 0.23a	0.58 ± 0.14abc	0.36 ± 0.1bc	***	*	ns
							-P	0.78 ± 0.48ab	0.19 ± 0.15c	0.39 ± 0.21bc			
Carbonyl-1P	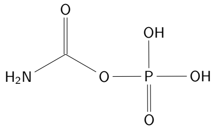	156	18	2.8	590-55-6	YS	+P	1 ± 0.07d	1.88 ± 0.02a	1.23 ± 0.04c	***	***	***
							-P	0.94 ± 0.03d	1.39 ± 0.06b	0.76 ± 0.05e			
						Leaves	+P	1 ± 0.2e	2.58 ± 0.11b	1.82 ± 0.06d	***	***	***
							-P	2.29 ± 0.07c	3.16 ± 0.13a	2.12 ± 0.02c			
						Roots	+P	1 ± 0.22c	1.92 ± 0.01a	1.42 ± 0.09b	***	***	***
							-P	0.44 ± 0.16e	1.71 ± 0.09a	0.73 ± 0.12d			
N-Acetyl-L-Citrulline	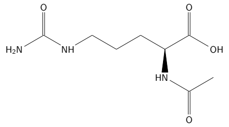	112	22	3.55	33965-42-3	YS	+P	1 ± 0.07d	1.85 ± 0.02a	1.21 ± 0.04c	***	***	***
							-P	0.94 ± 0.03d	1.38 ± 0.06b	0.75 ± 0.05e			
						Leaves	+P	1 ± 0.2e	2.58 ± 0.11b	1.82 ± 0.06d	***	***	***
							-P	2.29 ± 0.07c	3.16 ± 0.13a	2.12 ± 0.02c			
						Roots	+P	1 ± 0.12ab	1.13 ± 0.15a	0.89 ± 0.04bc	***	***	***
							-P	1.14 ± 0.17a	0.53 ± 0.03d	0.79 ± 0.1c			

Continue on the next page

Light intensity and P interaction effect on Primary metabolites and/or related anaplerotic pathway metabolites to carbohydrates and amino acids of Longjing-43 cultivar measured by GC×GC-TOF/MS analysis (Data normalise to 1 based on treatment (FL+P) (*cont.*).

Metabolites	Chemical Structure	Mass	1st Dim Time (Minutes)	2nd Dim Time (Seconds)	CAS	Organ	P level	Light Intensity			Significance		
								Full Light (FL)	Medium Light (ML)	Low Light (LL)	FL	ML	LL
Citrulline	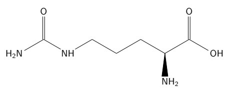	70	13.9	2.23	372-75-8	YS	+P	1 ± 0.06d	1.83 ± 0.02a	1.19 ± 0.04c	***	***	***
							-P	0.93 ± 0.03d	1.37 ± 0.06b	0.74 ± 0.05e			
						Leaves	+P	1 ± 0.13a	0.46 ± 0.04d	0.85 ± 0.08b	***	***	***
							-P	0.71 ± 0.05c	0.19 ± 0.01e	0.16 ± 0.01e			
						Roots	+P	1 ± 0.21b	1.62 ± 0.25a	0.77 ± 0.1b	***	***	***
							-P	0.03 ± 0.01c	0.02 ± 0c	0.07 ± 0c			
L-Arginine	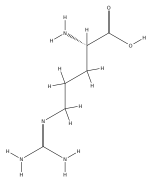	179	23.6	2.93	74-79-3	YS	+P	1 ± 0.03d	2.01 ± 0.02a	1.31 ± 0.04c	***	***	***
							-P	1.05 ± 0.08d	1.48 ± 0.07b	0.82 ± 0.05e			
						Leaves	+P	1 ± 0.03ab	0.75 ± 0c	0.68 ± 0.05c	***	**	***
							-P	0.94 ± 0.03b	1.08 ± 0.07a	0.27 ± 0.07d			
						Roots	+P	1 ± 0.02b	0.92 ± 0.01c	1.02 ± 0.01b	***	***	***
							-P	1.04 ± 0.01ab	1.09 ± 0.06a	1.09 ± 0.02a			
Ornithine	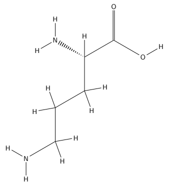	69	15.8	2.89	70-26-8	YS	+P	1 ± 0.04d	1.63 ± 0.03a	1.09 ± 0.03c	***	***	***
							-P	0.95 ± 0.03d	1.24 ± 0.07b	0.73 ± 0.05e			
						Leaves	+P	1 ± 0.03b	0.68 ± 0.02c	0.43 ± 0.07d	***	*	***
							-P	0.72 ± 0.07c	1.26 ± 0.09a	0.02 ± 0.01e			
						Roots	+P	1 ± 0.01ab	0.88 ± 0.01c	0.97 ± 0.01b	***	***	***
							-P	0.96 ± 0.02b	1.04 ± 0.05a	1.04 ± 0.02a			
Chorismate	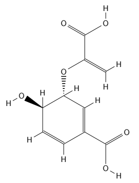	101	7.5	1.69	617-12-9	YS	+P	1 ± 0.06d	1.78 ± 0.02a	1.18 ± 0.03c	***	***	***
							-P	0.9 ± 0.03e	1.47 ± 0.07b	0.67 ± 0.05f			
						Leaves	+P	1 ± 0.03b	0.68 ± 0.02c	0.44 ± 0.07d	***	ns	***
							-P	0.73 ± 0.07c	1.26 ± 0.09a	0.04 ± 0.01e			
						Roots	+P	1 ± 0.14ab	1.12 ± 0.16a	0.83 ± 0.05bc	***	***	***
							-P	1.2 ± 0.21a	0.38 ± 0.03d	0.69 ± 0.12c			

Continue on the next page

Light intensity and P interaction effect on Primary metabolites and/or related anaplerotic pathway metabolites to carbohydrates and amino acids of Longjing-43 cultivar measured by GC×GC-TOF/MS analysis (Data normalise to 1 based on treatment (FL+P) (*cont.*).

Metabolites	Chemical Structure	Mass	1st Dim Time (Minutes)	2nd Dim Time (Seconds)	CAS	Organ	P level	Light Intensity: Full Light (FL)	Medium Light (ML)	Low Light (LL)	Significance: FL	ML	LL
Anthranilate	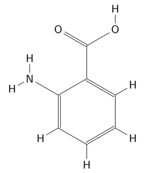	146	10.5	1.72	118-92-3	YS	+P	1 ± 0.06d	2.24 ± 0.04a	1.46 ± 0.04b	***	***	***
							-P	1.05 ± 0.03d	1.28 ± 0.06c	0.8 ± 0.06e			
						Leaves	+P	1 ± 0.07bc	1.28 ± 0.31a	0.72 ± 0.06c	***	**	*
							-P	0.91 ± 0.03c	1.25 ± 0.2ab	0.34 ± 0.09d			
						Roots	+P	1 ± 0.18a	1.05 ± 0.07a	0.73 ± 0.05b	***	***	***
							-P	1.11 ± 0.04a	0.21 ± 0.01d	0.54 ± 0.07c			
Prephenate	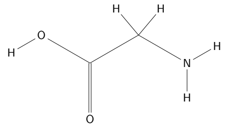	45	31.8	4.38	126-49-8	YS	+P	1 ± 0.03d	2.2 ± 0.04a	1.43 ± 0.04b	***	***	***
							-P	0.95 ± 0.06d	1.25 ± 0.06c	0.74 ± 0.06e			
						Leaves	+P	1 ± 0.18a	0.16 ± 0d	0.63 ± 0.01c	***	***	***
							-P	0.7 ± 0.04c	0.85 ± 0.09b	0.56 ± 0.03c			
						Roots	+P	1 ± 0.08b	2 ± 0.08a	0.99 ± 0.02b	***	***	***
							-P	0.09 ± 0.07cd	0.14 ± 0.03c	0.04 ± 0.01d			
L-Arogenate	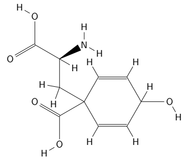	218	19.3	3.29	53078-86-7	YS	+P	1 ± 0.05d	1.76 ± 0.02a	1.18 ± 0.04c	***	***	***
							-P	0.91 ± 0.03e	1.48 ± 0.07b	0.69 ± 0.05f			
						Leaves	+P	1 ± 0.05a	0.92 ± 0.01b	0.77 ± 0.05c	***	***	***
							-P	0.84 ± 0.02c	0.6 ± 0.03d	0.3 ± 0.06e			
						Roots	+P	1 ± 0.03c	1.09 ± 0.03b	1.04 ± 0.03c	***	***	***
							-P	0.91 ± 0.04d	1.17 ± 0.01a	0.94 ± 0.01d			
L-Theanine	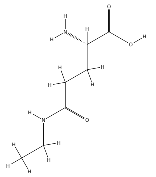	46	19	3.91	3081-61-6	YS	+P	1 ± 0.04c	1.79 ± 0.02a	1.19 ± 0.03b	***	***	***
							-P	0.96 ± 0.03c	1.25 ± 0.06b	0.73 ± 0.06d			
						Leaves	+P	1 ± 0.01a	0.91 ± 0.01b	0.54 ± 0.05c	***	***	***
							-P	0.44 ± 0.03d	0.15 ± 0.05f	0.24 ± 0.06e			
						Roots	+P	1 ± 0.08c	0.35 ± 0.1e	0.85 ± 0.05d	***	***	***
							-P	1.56 ± 0.01a	1.66 ± 0.01a	1.38 ± 0.05b			

Means with the different letter in the row of the same metabolites content are significantly different.
'***' 0.001 '**' 0.01 '*' 0.05 = significant differences, ns = non-significant differences, between light and P interaction.

Table S7. Identification of secondary metabolites measured by UPLC–Q–TOF/MS and ANOVA analysis in tea plant organs in response light effect, P effect and their interaction.

Metabolites	Chemical Structure	Mass	RT	Adduct	Organ	Plevel	Light Intensity Full Light (FL)	Medium Light (ML)	Low Light (LL)	Significance FL	ML	LL
p–Coumaroyl shikimic acid	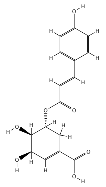	320.089602866	3.73	M-H	YS	+P	1 ± 0.15d	3.69 ± 0.87c	10.19 ± 0.45a	***	***	***
						-P	5.99 ± 0.36b	2.59 ± 0.97c	3.55 ± 0.79c			
					Leaves	+P	1 ± 0.16b	2.19 ± 1.71b	2.01 ± 0.07b	***	***	***
						-P	5.04 ± 3.78b	11.94 ± 4.69a	1.25 ± 0.08b			
					Roots	+P	1 ± 0.07a	0.95 ± 0.04a	0.74 ± 0.05b	***	***	***
						-P	0.06 ± 0.01d	0.6 ± 0.08c	0.98 ± 0.11a			
p–Coumaroyl quinic acid	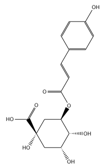	338.100167552	4.48	M-H	YS	+P	1 ± 0.14e	13.82 ± 0.44a	5.01 ± 1.13c	***	***	***
						-P	3.45 ± 0.45d	1.17 ± 0.82e	8.28 ± 1.33b			
					Leaves	+P	1 ± 0.61c	43.88 ± 39.99ab	0.27 ± 0.03c	***	**	ns
						-P	24.9 ± 13.8abc	58.46 ± 14.26a	21.71 ± 20.82bc			
					Roots	+P	1 ± 0.02c	1.85 ± 0.27b	1.95 ± 0.18b	***	ns	***
						-P	0.27 ± 0.09d	3.18 ± 0.31a	0.93 ± 0.25c			
Caffeoyl shikimic acid	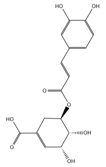	336.084517488	6.46	M-H2O-H	YS	+P	1 ± 0.21a	0.12 ± 0c	0.24 ± 0.05c	***	*	**
						-P	0.75 ± 0.06ab	0.44 ± 0.34bc	0.7 ± 0.46ab			
					Leaves	+P	1 ± 0.21a	10.78 ± 14.27a	2.18 ± 0.08a	*	ns	ns
						-P	0.28 ± 0.03a	7.09 ± 9.12a	1.42 ± 0.41a			
					Roots	+P	1 ± 0.13b	1.46 ± 0.11a	1.04 ± 0.09b	***	***	***
						-P	0.06 ± 0.03d	1.34 ± 0.13a	0.74 ± 0.07c			
Caffeoyl quinic acid	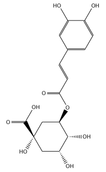	354.095082174	4.91	M-H2O-H	YS	+P	1 ± 0.1ab	0.24 ± 0e	0.44 ± 0.02d	***	***	***
						-P	0.62 ± 0.03c	0.87 ± 0.05b	1.09 ± 0.17a			
					Leaves	+P	1 ± 0.19a	2.46 ± 2.59a	0.61 ± 0.02a	**	ns	ns
						-P	2.57 ± 0.35a	2.87 ± 2.3a	0.5 ± 0.02a			
					Roots	+P	1 ± 0.16b	0.51 ± 0.03cd	0.66 ± 0.15c	***	***	ns
						-P	0.01 ± 0.01e	0.39 ± 0.08d	1.61 ± 0.19a			

Continue on the next page

Identification of secondary metabolites measured by UPLC–Q–TOF/MS and ANOVA analysis in tea plant organs in response light effect, P effect and their interaction (*cont.*).

Metabolites	Chemical Structure	Mass	RT	Adduct	Organ	Plevel	Light Intensity: Full Light (FL)	Light Intensity: Medium Light (ML)	Light Intensity: Low Light (LL)	Significance: FL	Significance: ML	Significance: LL
Caffeoyl–CoA	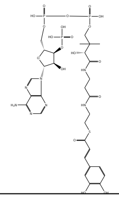	929.146902423	2.96	M-H	YS	+P	1 ± 0.05e	4.8 ± 0.11d	9.21 ± 0.75c	***	***	***
						-P	2.5 ± 0.25e	11.35 ± 0.86b	16.14 ± 2.66a			
					Leaves	+P	1 ± 0.27b	19.34 ± 18.84a	1.64 ± 0.26b	*	*	ns
						-P	1.26 ± 0.98b	5.83 ± 2.48ab	8.94 ± 9.34ab			
					Roots	+P	1 ± 0.05b	1.16 ± 0.05b	1.3 ± 0.17b	**	***	***
						-P	1.68 ± 0.15a	1.68 ± 0.12a	0.99 ± 0.37b			
Quercetin	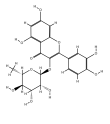	448.100561482	3.32	M-H	YS	+P	1 ± 0.23e	16.03 ± 0.75b	7.1 ± 1.43c	***	***	*
						-P	4.4 ± 0.71d	18.89 ± 1.96a	8.1 ± 0.53c			
					Leaves	+P	1 ± 0.44c	1.95 ± 0.55c	2.45 ± 0.91bc	*	***	***
						-P	4.78 ± 1.95a	4.38 ± 1.83ab	1.04 ± 0.05c			
					Roots	+P	1 ± 0.1cd	1.31 ± 0.12c	1.01 ± 0.11cd	***	***	***
						-P	0.71 ± 0.4d	1.77 ± 0.13b	2.15 ± 0.18a			
Isoquercetin	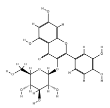	464.0954761	4.85	M-H	YS	+P	1 ± 0.05b	0.21 ± 0.03e	0.77 ± 0.08c	***	***	***
						-P	1.17 ± 0.05a	0.65 ± 0.07d	1.08 ± 0.04ab			
					Leaves	+P	1 ± 0.29c	1.09 ± 0.26c	1.55 ± 0.26b	***	***	ns
						-P	1.37 ± 0.19bc	0.37 ± 0.31d	1.99 ± 0.07a			
					Roots	+P	1 ± 0.1c	1.72 ± 0.09ab	1.01 ± 0.28c	***	***	**
						-P	1.2 ± 0.21c	1.94 ± 0.07a	1.62 ± 0.12b			
Rutin	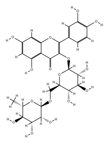	610.1533849	4.5	M-H2O-H	YS	+P	1 ± 0.14b	3.78 ± 0.09a	1.43 ± 0.34b	***	*	***
						-P	0.46 ± 0.05b	0.11 ± 0.06b	3.76 ± 1.87a			
					Leaves	+P	1 ± 0.15b	1.8 ± 0.76b	1.66 ± 0.06b	***	***	***
						-P	3.86 ± 0.15a	4.69 ± 1.46a	1.08 ± 0.04b			
					Roots	+P	1 ± 0.09d	3.65 ± 0.73b	2.24 ± 1.22c	***	**	***

Continue on the next page

Identification of secondary metabolites measured by UPLC–Q–TOF/MS and ANOVA analysis in tea plant organs in response light effect, P effect and their interaction (*cont.*).

Metabolites	Chemical Structure	Mass	RT	Adduct	Organ	Plevel	Light Intensity			Significance		
							Full Light (FL)	Medium Light (ML)	Low Light (LL)	FL	ML	LL
						-P	0.24 ± 0.02d	8.19 ± 0.87a	0.99 ± 0.36d			
Leucocyanidin	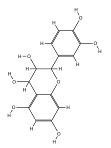	306.073952802	2.53	M+FA+H	YS	+P	1 ± 0.08a	0.06 ± 0d	0.06 ± 0.01d	***	***	***
						-P	0.51 ± 0.02b	0.46 ± 0.03b	0.38 ± 0.01c			
					Leaves	+P	1 ± 0.25a	3.02 ± 2.58a	0.93 ± 0.05a	ns	ns	ns
						-P	2.16 ± 0.36a	2.99 ± 4.28a	2.94 ± 0.02a			
					Roots	+P	1 ± 0.07cd	1.94 ± 0.41b	1.58 ± 0.57bc	***	***	***
						-P	0.56 ± 0.05d	4.5 ± 0.44a	1.35 ± 0.23bc			
Cyanidin	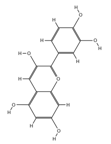	286.0477381	3.16	2H/M+FA-H	YS	+P	1 ± 0.23b	0.01 ± 0d	0.26 ± 0.15c	***	***	***
						-P	1.29 ± 0.12a	0.22 ± 0.14cd	1.17 ± 0.07ab			
					Leaves	+P	1 ± 0.29b	1.52 ± 0.36b	1.03 ± 0.02b	***	***	***
						-P	4.68 ± 0.29a	3.67 ± 2.15a	1.24 ± 0.17b			
					Roots	+P	1 ± 0.13a	0.91 ± 0.03a	0.97 ± 0.11a	***	***	***
						-P	0.04 ± 0.02b	1.04 ± 0.11a	0.15 ± 0.06b			
(+)–Catechin	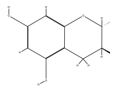	289.0741	2.38	M-H 2.26	YS	+P	1 ± 0.18b	0.04 ± 0c	0.04 ± 0.01c	***	**	**
						-P	1.5 ± 0.35a	0.06 ± 0.01c	0.09 ± 0.01c			
					Leaves	+P	1 ± 0.17b	1.78 ± 0.72b	1.64 ± 0.06b	***	***	***
						-P	3.8 ± 0.26a	4.32 ± 1.62a	1.07 ± 0.04b			
					Roots	+P	1 ± 0.09b	1.61 ± 0.04a	1.17 ± 0.18b	***	***	***
						-P	0.68 ± 0.04c	1.54 ± 0.1a	0.54 ± 0.07c			
Cyanidin 3–glucoside	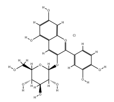	448.100561482	11.66	M-H	YS	+P	1 ± 0.14bc	1.71 ± 0.07a	0.73 ± 0.09c	*	**	***
						-P	0.79 ± 0.1bc	0.66 ± 0.53c	1.19 ± 0.18b			
					Leaves	+P	1 ± 0.07ab	1.1 ± 0.41a	0.28 ± 0.06c	*	***	ns
						-P	0.64 ± 0.12abc	0.52 ± 0.55bc	0.81 ± 0.07ab			
					Roots	+P	1 ± 0.16ab	1.15 ± 0.13a	0.83 ± 0.15bc	***	***	***
						-P	0.07 ± 0.02e	0.61 ± 0.07d	0.67 ± 0.04cd			

Continue on the next page

Identification of secondary metabolites measured by UPLC–Q–TOF/MS and ANOVA analysis in tea plant organs in response light effect, P effect and their interaction (*cont.*).

Metabolites	Chemical Structure	Mass	RT	Adduct	Organ	Plevel	Light Intensity: Full Light (FL)	Light Intensity: Medium Light (ML)	Light Intensity: Low Light (LL)	Significance: FL	Significance: ML	Significance: LL
Cyanidin 3–rutinoside	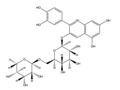	204	21.5	2.26	YS	+P	1 ± 0.22a	0.14 ± 0.01c	0.12 ± 0.01c	***	***	**
						-P	0.71 ± 0.07b	0.04 ± 0.04c	0.14 ± 0.04c			
					Leaves	+P	1 ± 0.02bc	1.48 ± 0.96bc	0.58 ± 0.03c	***	***	***
						-P	2.43 ± 1.73b	6.54 ± 1.57a	0.63 ± 0.06bc			
					Roots	+P	1 ± 0.08b	1.53 ± 0.07a	0.91 ± 0.17b	***	***	***
						-P	0.15 ± 0.01c	1.46 ± 0.14a	0.83 ± 0.04b			
Procyanidin B1	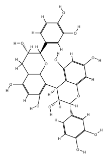	578.1424263	2.62	M-H2O-H	YS	+P	1 ± 0.02a	0.53 ± 0.29bc	0.36 ± 0.04cd	***	***	*
						-P	0.66 ± 0.03b	0.1 ± 0.02e	0.24 ± 0.05de			
					Leaves	+P	1 ± 0.59ab	4.93 ± 6.04a	0.43 ± 0.02b	**	ns	ns
						-P	4.55 ± 1.09ab	0.82 ± 1.02ab	3.27 ± 0.01ab			
					Roots	+P	1 ± 0.08e	0.62 ± 0.16f	1.53 ± 0.11d	***	***	***
						-P	3.69 ± 0.17a	1.89 ± 0.2c	2.51 ± 0.03b			
(-)–Epicatechin	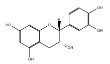	290.07903818	3.32	M-H2O-H	YS	+P	1 ± 0.23a	0.04 ± 0c	0.02 ± 0c	***	**	***
						-P	0.67 ± 0.12b	0.01 ± 0.01c	0.02 ± 0c			
					Leaves	+P	1 ± 0.06bc	1.46 ± 0.85b	0.59 ± 0.02cd	***	**	***
						-P	0.1 ± 0.07d	0.18 ± 0.14d	3.92 ± 0.04a			
					Roots	+P	1 ± 0.07b	0.94 ± 0.04bc	0.84 ± 0.07c	***	**	***
						-P	0.79 ± 0.12c	0.62 ± 0.07d	1.67 ± 0.12a			
Myricetin	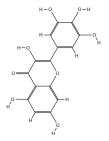	318.037567296	4.4	M+FA-H	YS	+P	1 ± 0.27c	3.33 ± 0.71a	3.43 ± 0.43a	***	***	***
						-P	2.39 ± 0.25b	0 ± 0d	1.8 ± 0.48b			
					Leaves	+P	1 ± 0.94ab	0.46 ± 0.24bc	1.87 ± 0.79a	***	***	ns
						-P	0.21 ± 0.05bc	0.03 ± 0.01c	0.64 ± 0.19bc			
					Roots	+P	1 ± 0.01bc	1.34 ± 0.25b	1.12 ± 0.37bc	***	***	***
						-P	0.78 ± 0.05cd	2.89 ± 0.24a	0.57 ± 0.11d			

Continue on the next page

Identification of secondary metabolites measured by UPLC–Q–TOF/MS and ANOVA analysis in tea plant organs in response light effect, P effect and their interaction (*cont.*).

Metabolites	Chemical Structure	Mass	RT	Adduct	Organ	Plevel	Light Intensity			Significance		
							Full Light (FL)	Medium Light (ML)	Low Light (LL)	FL	ML	LL
Leucodelphinidin	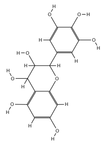	322.068867424	2.53	M+FA-H	YS	+P	1 ± 0.09a	0.64 ± 0.02c	0.43 ± 0.02d	***	*	***
						-P	0.65 ± 0.01c	0.81 ± 0.07b	0.74 ± 0.08bc			
					Leaves	+P	1 ± 0.12c	1.86 ± 0.57abc	1.69 ± 0.07bc	*	**	***
						-P	2.19 ± 0.37ab	1.28 ± 1.21bc	2.74 ± 0.05a			
					Roots	+P	1 ± 0.07c	1.93 ± 0.11a	0.95 ± 0.2c	***	***	***
						-P	0.17 ± 0.03d	1.26 ± 0.12b	1.05 ± 0.09c			
Delphinidin	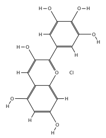	337.0149	3.9	M-H	YS	+P	1 ± 0.15b	0.19 ± 0.02c	0.15 ± 0.03c	***	***	**
						-P	2.36 ± 0.34a	0.59 ± 0.26bc	0.79 ± 0.56b			
					Leaves	+P	1 ± 0.9ab	0.3 ± 0.34bc	1.9 ± 0.83a	***	**	*
						-P	0.98 ± 0.23ab	0.01 ± 0.01c	0.59 ± 0.34bc			
					Roots	+P	1 ± 0.02e	2.32 ± 0.5c	1.48 ± 0.94de	***	***	***
						-P	4.07 ± 0.11b	5.45 ± 0.12a	2.23 ± 0.2cd			
(+)-Gallocatechin	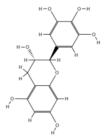	306.0739528	2.04	M-H2O-H	YS	+P	1 ± 0.06a	0.44 ± 0.01d	0.21 ± 0.01e	***	***	ns
						-P	0.63 ± 0.03b	0.52 ± 0.02c	0.5 ± 0.01c			
					Leaves	+P	1 ± 0.58b	1 ± 0.18b	2.08 ± 0.58a	***	***	ns
						-P	0.19 ± 0.07c	0.51 ± 0.65bc	0.77 ± 0.18bc			
					Roots	+P	1 ± 0.03a	0.76 ± 0.05b	0.72 ± 0.08b	***	***	***
						-P	0.03 ± 0.01e	0.47 ± 0.05c	0.34 ± 0.02d			
Delphinidin 3–glucoside	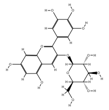	464.095476104	4.86	M-H2O-H	YS	+P	1 ± 0.22a	0.09 ± 0c	0.1 ± 0c	***	**	**
						-P	0.7 ± 0.07b	0.04 ± 0.04c	0.12 ± 0.05c			
					Leaves	+P	1 ± 0.04c	6.29 ± 2.7b	0.45 ± 0.04c	***	***	***
						-P	0.52 ± 0.32c	0.26 ± 0.22c	16.64 ± 0.72a			
					Roots	+P	1 ± 0.11ab	1.11 ± 0.09a	0.68 ± 0.03c	***	***	***
						-P	0.16 ± 0.03e	0.48 ± 0.04d	0.98 ± 0.1b			

Continue on the next page

Identification of secondary metabolites measured by UPLC–Q–TOF/MS and ANOVA analysis in tea plant organs in response light effect, P effect and their interaction (*cont.*).

Metabolites	Chemical Structure	Mass	RT	Adduct	Organ	Plevel	Light Intensity			Significance		
							Full Light (FL)	Medium Light (ML)	Low Light (LL)	FL	ML	LL
(-)–Epigallocatechin	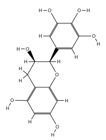	306.0739528	2.72	M+FA-H	YS	+P	1 ± 0.22a	0.08 ± 0.01d	0.25 ± 0.01c	***	***	ns
						-P	0.78 ± 0.06b	0.04 ± 0d	0.05 ± 0.03d			
					Leaves	+P	1 ± 0.7ab	0.81 ± 0.36abc	1.4 ± 0.61a	*	***	ns
						-P	0.12 ± 0.05c	0.32 ± 0.16bc	0.66 ± 0.12bc			
					Roots	+P	1 ± 0.12b	2.35 ± 0.21a	0.85 ± 0.26b	***	***	***
						-P	0.02 ± 0c	0.86 ± 0.09b	0.28 ± 0.04c			
Kaempeferol	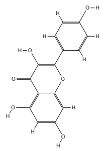	286.047738052	3.34	M-H	YS	+P	1 ± 0.19a	0.02 ± 0d	0.01 ± 0d	***	***	ns
						-P	0.42 ± 0.02b	0.32 ± 0.01bc	0.28 ± 0.02c			
					Leaves	+P	1 ± 0.5bc	1.25 ± 0.16abc	1.5 ± 0.4ab	*	***	ns
						-P	1.75 ± 0.27a	0.81 ± 0.33c	0.71 ± 0.04c			
					Roots	+P	1 ± 0.14b	0.51 ± 0.01d	0.74 ± 0.12c	***	***	ns
						-P	0.09 ± 0e	0.35 ± 0.03d	1.64 ± 0.18a			
Malvidin	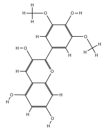	330.047/ 366.050630541	0.81	M-H/M-H2O-H	YS	+P	1 ± 0b	0.01 ± 0e	0.12 ± 0.01de	***	***	***
						-P	1.87 ± 0a	0.17 ± 0.01cd	0.24 ± 0.17c			
					Leaves	+P	1 ± 0.35ab	0.9 ± 0.14ab	1.29 ± 0.32a	ns	***	ns
						-P	0.47 ± 0.08c	0.67 ± 0.28bc	0.62 ± 0.09bc			
					Roots	+P	1 ± 0.1d	0.53 ± 0.1e	1.23 ± 0.09c	***	***	***
						-P	2.07 ± 0.08b	1.29 ± 0.09c	2.33 ± 0.09a			
Luteolin	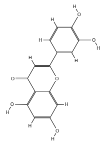	286.047738052	2.85	M-H	YS	+P	1 ± 0.13a	0.01 ± 0c	0.02 ± 0c	***	***	***
						-P	0.6 ± 0.07b	0.02 ± 0c	0.01 ± 0c			
					Leaves	+P	1 ± 0ab	1.29 ± 1.32a	0.15 ± 0.06b	ns	***	*
						-P	0.07 ± 0.04b	0.12 ± 0.1b	0.29 ± 0.03b			
					Roots	+P	1 ± 0.04a	0.94 ± 0.04a	0.69 ± 0.06b	***	***	***
						-P	0.27 ± 0.15c	0.62 ± 0.05b	0.95 ± 0.1a			

Continue on the next page

Identification of secondary metabolites measured by UPLC–Q–TOF/MS and ANOVA analysis in tea plant organs in response light effect, P effect and their interaction (*cont.*).

Metabolites	Chemical Structure	Mass	RT	Adduct	Organ	Plevel	Light Intensity: Full Light (FL)	Light Intensity: Medium Light (ML)	Light Intensity: Low Light (LL)	Significance: FL	Significance: ML	Significance: LL
Apigenin	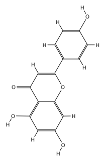	270.05282343	3.64	3.64	YS	+P	1 ± 0.13a	0.07 ± 0c	0.06 ± 0c	***	***	***
						-P	0.6 ± 0.07b	0.01 ± 0c	0.04 ± 0c			
					Leaves	+P	1 ± 0.02ab	0.77 ± 0.39abc	0.15 ± 0.06c	***	**	ns
						-P	0.49 ± 0.29bc	1.24 ± 0.78a	0.43 ± 0.03bc			
					Roots	+P	1 ± 0.01b	1.52 ± 0.07a	0.8 ± 0.1c	***	***	***
						-P	0.12 ± 0.02d	0.87 ± 0.09c	0.76 ± 0.07c			
Luteolin 7–*O* glucoside	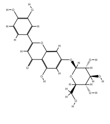	462.07982604	3.7	M+FA-H	YS	+P	1 ± 0.22c	4.83 ± 0.47a	1.51 ± 0.37c	***	***	***
						-P	1.53 ± 0.17c	0.04 ± 0d	2.68 ± 0.43b			
					Leaves	+P	1 ± 0.37b	0.51 ± 0.1c	2.07 ± 0.11a	***	***	***
						-P	0.06 ± 0.04d	0.27 ± 0.37cd	0.6 ± 0.21bc			
					Roots	+P	1 ± 0.13b	1.86 ± 0.16a	0.73 ± 0.14c	***	***	***
						-P	0.2 ± 0.09d	0.64 ± 0.04c	0.38 ± 0.02d			
Tricin 7–glucuronide	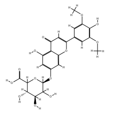	1034.217561	3.83	M-H	YS	+P	1 ± 0.16b	0.19 ± 0.12cd	1.13 ± 0.06ab	***	***	***
						-P	1.21 ± 0.11a	0.01 ± 0.01d	0.22 ± 0.13c			
					Leaves	+P	1 ± 0.02a	0.9 ± 0.25a	0.52 ± 0.04bc	***	***	ns
						-P	0.44 ± 0.08c	0.32 ± 0.32c	0.79 ± 0.01ab			
					Roots	+P	1 ± 0.01bc	1.41 ± 0.27b	1.17 ± 0.42bc	***	***	***
						-P	0.85 ± 0.05cd	3.13 ± 0.26a	0.47 ± 0.13d			
Shikimic acid	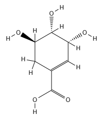	174.0528234	3.43	M-H	YS	+P	1 ± 0.12e	12.06 ± 0.65c	17.13 ± 0.36b	***	***	***
						-P	8.84 ± 0.29d	12.84 ± 0.46c	21.74 ± 1.69a			
					Leaves	+P	1 ± 0.04a	0.78 ± 0.22ab	0.23 ± 0.09c	***	***	***
						-P	0.37 ± 0.08c	0.14 ± 0.24c	0.68 ± 0.03b			
					Roots	+P	1 ± 0.13c	0.49 ± 0.08d	1.09 ± 0.11c	***	***	***
						-P	1.33 ± 0.04b	1.02 ± 0.05c	2.25 ± 0.13a			

Continue on the next page

Identification of secondary metabolites measured by UPLC–Q–TOF/MS and ANOVA analysis in tea plant organs in response light effect, P effect and their interaction (*cont.*).

Metabolites	Chemical Structure	Mass	RT	Adduct	Organ	Plevel	Light Intensity: Full Light (FL)	Light Intensity: Medium Light (ML)	Light Intensity: Low Light (LL)	Significance: FL	Significance: ML	Significance: LL
m–trigallic acid	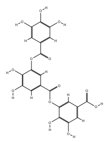	474.0434405	3.32	M-H2O-H	YS	+P	1 ± 0.07c	0.71 ± 0.02d	1.55 ± 0.14b	***	***	***
						-P	0.36 ± 0.03e	0.96 ± 0.03c	6.4 ± 0.29a			
					Leaves	+P	1 ± 0.28b	0.65 ± 0.32bc	0.34 ± 0.13c	***	**	***
						-P	2.05 ± 0.27a	0.38 ± 0.08c	0.29 ± 0.04c			
					Roots	+P	1 ± 0.04b	1.4 ± 0.09a	0.77 ± 0.06c	***	***	***
						-P	0.26 ± 0.06e	0.63 ± 0.05d	0.98 ± 0.08b			
quinic acid	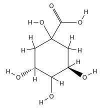	192.0633881	0.81	M-H2O-H	YS	+P	1 ± 0.31d	50.73 ± 5.6a	42.71 ± 16.68a	***	*	***
						-P	21.11 ± 7.03bc	12.31 ± 9.1cd	36.49 ± 16.06ab			
					Leaves	+P	1 ± 0.39ab	0.8 ± 0.11ab	1.25 ± 0.35a	**	***	ns
						-P	0.07 ± 0.06d	0.24 ± 0.4cd	0.57 ± 0.1bc			
					Roots	+P	1 ± 0.18bc	0.64 ± 0.1d	0.75 ± 0.16cd	***	**	***
						-P	0.01 ± 0e	1.03 ± 0.21b	1.91 ± 0.21a			
2,4-hydrobenzoic acid	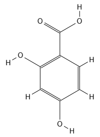	154.0266087	3.31	M-H2O-H	YS	+P	1 ± 0.15c	7.7 ± 2.21a	3.81 ± 1.14b	***	***	***
						-P	2.39 ± 0.67bc	0.43 ± 0.26c	4.04 ± 1.63b			
					Leaves	+P	1 ± 0.7ab	1.1 ± 0.2ab	1.97 ± 0.92a	*	***	ns
						-P	0.13 ± 0.09b	0.53 ± 0.85b	0.53 ± 0.08b			
					Roots	+P	1 ± 0.09cd	2 ± 0.17a	0.79 ± 0.17d	***	***	ns
						-P	1.64 ± 0.07b	0.82 ± 0.1d	1.14 ± 0.11c			
4′–methylchalcone	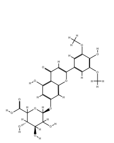	534.1502	2.62	M-H2O-H	YS	+P	1 ± 0.09c	39.54 ± 18.01a	22.89 ± 7.3ab	**	**	***
						-P	12.11 ± 4.36bc	0.13 ± 0.01c	19.96 ± 12.84b			
					Leaves	+P	1 ± 0.26ab	0.7 ± 0.14bc	1.13 ± 0.33a	***	***	ns
						-P	0.06 ± 0.04d	0.09 ± 0.12d	0.39 ± 0.03cd			
					Roots	+P	1 ± 0.14b	1.65 ± 0.17a	0.7 ± 0.1c	***	***	***
						-P	0.1 ± 0e	0.42 ± 0.04d	0.08 ± 0.03e			

Continue on the next page

Identification of secondary metabolites measured by UPLC–Q–TOF/MS and ANOVA analysis in tea plant organs in response light effect, P effect and their interaction (*cont.*).

Metabolites	Chemical Structure	Mass	RT	Adduct	Organ	Plevel	Light Intensity			Significance		
							Full Light (FL)	Medium Light (ML)	Low Light (LL)	FL	ML	LL
4,2′,4′,alpha-tetra-hydroxydihydrochalcone	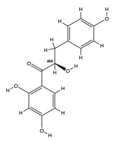	274.0841236	4.13	M+Na-2H	YS	+P	1 ± 0.06d	3.59 ± 0.25c	22.11 ± 1.28a	***	ns	***
						-P	12.04 ± 0.72b	1.45 ± 0.12d	11.64 ± 1.3b			
					Leaves	+P	1 ± 0.16bc	0.67 ± 0.26c	1.74 ± 0.11a	***	***	ns
						-P	1.85 ± 0.37a	0.19 ± 0.31d	1.42 ± 0.17ab			
					Roots	+P	1 ± 0c	0.54 ± 0.15d	0.77 ± 0.23cd	***	***	***
						-P	3.51 ± 0.29a	1.71 ± 0.2b	1.41 ± 0.17b			
Prunin 6′ P-coumarate	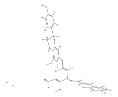	580.1580764	2.97	M-H2O-H	YS	+P	1 ± 0.07c	0.63 ± 0.13d	1.74 ± 0.21a	***	***	***
						-P	1.32 ± 0.09b	0.39 ± 0.05e	0.8 ± 0.05cd			
					Leaves	+P	1 ± 0.01a	0.5 ± 0.01b	0.18 ± 0.07d	***	***	***
						-P	0.06 ± 0.05e	0.01 ± 0.01e	0.3 ± 0.03c			
					Roots	+P	1 ± 0.02b	0.93 ± 0.08b	0.87 ± 0.23b	***	***	***
						-P	0.08 ± 0.08c	1.4 ± 0.14a	0.27 ± 0.01c			
Naringenin	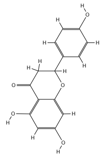	272.068473494	5.05	M-H	YS	+P	1 ± 0.22ab	1.39 ± 0.49a	1.44 ± 0.21a	***	***	***
						-P	1.36 ± 0.12a	0.02 ± 0c	0.78 ± 0.35b			
					Leaves	+P	1 ± 0.06a	0.54 ± 0.02b	0.29 ± 0.02c	***	***	***
						-P	0.06 ± 0.04d	0.03 ± 0.05d	0.3 ± 0.03c			
					Roots	+P	1 ± 0.02b	1.68 ± 0.13a	0.8 ± 0.13c	***	***	***
						-P	0 ± 0d	0.69 ± 0.07c	0.8 ± 0.08c			
Eriodictyol	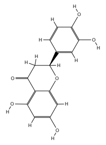	288.063388116	4.07	M-H	YS	+P	1 ± 0.2d	1.27 ± 0.14c	2.63 ± 0.14a	***	***	***
						-P	2.04 ± 0.11b	0.04 ± 0.01e	0.9 ± 0.18d			
					Leaves	+P	1 ± 0.04a	0.53 ± 0.01b	0.23 ± 0.03d	***	***	***
						-P	0.05 ± 0.05e	0.02 ± 0.03e	0.29 ± 0.03c			
					Roots	+P	1 ± 0.06b	1.44 ± 0.14a	0.71 ± 0.07c	***	***	***
						-P	0 ± 0e	0.38 ± 0.04d	0.66 ± 0.07c			

Means with the different letter in the row of the same metabolites content are significantly different.
‘***’ 0.001 ‘**’ 0.01 ‘*’ 0.05 = significant differences, ns = non-significant differences, between light and P interaction.

Table S8. Targeted metabolites amino acid and catechins (mg g^{-1}) in young shoots of tea plants in response light effect, P effect and their interaction effect.

Metabolites	P level	Light Intensity			Significance		
		FL	ML	LL	light	Plevel	L×P
Amino Acids							
Gly	+P	0.28 ± 0.07c	0.23 ± 0.09c	0.69 ± 0.12b	$p< 0.001$	$p< 0.001$	$p< 0.001$
	-P	1.35 ± 0.24a	0.79 ± 0.13b	0.19 ± 0.16c			
Thr	+P	0.56 ± 0.15c	1.68 ± 0.38a	0.47 ± 0.17c	$p< 0.001$	ns	$p< 0.001$
	-P	1.43 ± 0.21ab	1.3 ± 0.16b	0.39 ± 0.05c			
Ile	+P	0.15 ± 0.02bc	0.64 ± 0.14a	0.22 ± 0.03b	$p< 0.001$	$p< 0.001$	$p< 0.001$
	-P	0.26 ± 0.15b	0.19 ± 0.01bc	0.06 ± 0.02c			
Val	+P	0.06 ± 0.01bc	0.62 ± 0.15a	0.13 ± 0.01bc	$p< 0.001$	$p< 0.001$	$p< 0.001$
	-P	0.16 ± 0.04b	0.07 ± 0.01bc	0.03 ± 0c			
Phe	+P	0.2 ± 0.05c	1.38 ± 0.33a	0.61 ± 0.06b	$p< 0.001$	$p< 0.001$	$p< 0.001$
	-P	0.64 ± 0.06b	0.39 ± 0.05bc	0.18 ± 0.05c			
Ala	+P	0.09 ± 0a	0.02 ± 0.01d	0.06 ± 0.01bc	$p< 0.001$	$p< 0.001$	ns
	-P	0.04 ± 0.01c	0.11 ± 0.01a	0.07 ± 0.01b			
Asp	+P	2.59 ± 0.12b	0.74 ± 0.26d	1.3 ± 0.32c	$p< 0.001$	$p< 0.001$	$p< 0.001$
	-P	0.9 ± 0.09d	3.53 ± 0.2a	2.38 ± 0.25b			
Glu	+P	2.32 ± 0.07b	3.36 ± 0.93a	2.25 ± 0.23b	$p< 0.001$	ns	$p< 0.001$
	-P	3.36 ± 0.21a	3.75 ± 0.4a	1.65 ± 0.16b			
Pro	+P	1.25 ± 0.13e	7.68 ± 0.58a	2.33 ± 0.03d	$p< 0.001$	$p< 0.001$	$p< 0.001$
	-P	3.95 ± 0.41b	3.22 ± 0.61c	1.14 ± 0.33e			

Continue on the next page

Table S8. Targeted metabolites amino acid and catechins (mg g^{-1}) in young shoots of tea plants in response light effect, P effect and their interaction effect. (*cont.*).

Metabolites	P level	Light Intensity			Significance		
		FL	ML	LL	light	Plevel	L×P
Cys	+P	0.34 ± 0.04bc	0.11 ± 0.02d	0.25 ± 0.01c	$p< 0.05$	$p< 0.001$	$p< 0.001$
	-P	0.22 ± 0.01cd	0.59 ± 0.14a	0.41 ± 0.08b			
His	+P	0.4 ± 0.08b	0.24 ± 0.05c	0.07 ± 0.03d	$p< 0.001$	$p< 0.001$	$p< 0.01$
	-P	0.59 ± 0.04a	0.52 ± 0.08ab	0.49 ± 0.14ab			
Leu	+P	0.08 ± 0.01c	1.35 ± 0.22a	0.35 ± 0.08b	$p< 0.001$	$p< 0.001$	$p< 0.001$
	-P	0.5 ± 0.14b	0.08 ± 0.01c	0.05 ± 0.01c			
Lys	+P	0.92 ± 0.17b	0.29 ± 0.05d	0.5 ± 0.14cd	$p< 0.001$	$p< 0.001$	$p< 0.001$
	-P	0.42 ± 0.03d	0.67 ± 0.03c	1.35 ± 0.22a			
Met	+P	1.63 ± 0.08a	0.06 ± 0.02d	0.05 ± 0.01d	$p< 0.001$	$p< 0.001$	$p< 0.001$
	-P	0.13 ± 0.05cd	0.34 ± 0.24b	0.25 ± 0.06bc			
Tyr	+P	0.31 ± 0.05cd	1.43 ± 0.26a	0.63 ± 0.12b	$p< 0.001$	$p< 0.01$	$p< 0.001$
	-P	1.37 ± 0.12a	0.47 ± 0.03bc	0.14 ± 0.05d			
Catechins							
CG	+P	0.03 ± 0b	0.04 ± 0.01b	0.07 ± 0a	$p< 0.001$	$p< 0.001$	ns
	-P	0.08 ± 0.03a	0.03 ± 0b	0.04 ± 0b			
EC	+P	0.71 ± 0.02b	0.69 ± 0.05b	0.64 ± 0.03c	$p< 0.001$	ns	$p< 0.01$
	-P	0.78 ± 0.02a	0.69 ± 0.02b	0.62 ± 0.01c			
ECG	+P	2.28 ± 0.02c	2.74 ± 0.02b	2.17 ± 0.29c	$p< 0.001$	$p< 0.001$	ns
	-P	1.17 ± 0.01d	2.28 ± 0.45c	3.44 ± 0.26a			

Continue on the next page

Table S8. Targeted metabolites amino acid and catechins (mg g^{-1}) in young shoots of tea plants in response light effect, P effect and their interaction effect. (*cont.*).

Metabolites	P level	Light Intensity			Significance		
		FL	ML	LL	light	Plevel	L×P
GA	+P	0.65 ± 0.01a	0.54 ± 0.02c	0.63 ± 0.01a	$p < 0.001$	ns	$p < 0.001$
	-P	0.64 ± 0a	0.63 ± 0.01a	0.56 ± 0.01b			
GC	+P	0.15 ± 0.01a	0.15 ± 0.01a	0.15 ± 0.07a	ns	ns	ns
	-P	0.15 ± 0.03a	0.15 ± 0.04a	0.15 ± 0.04a			
GCG	+P	0.26 ± 0.02a	0.26 ± 0.03a	0.26 ± 0.04a	ns	ns	ns
	-P	0.3 ± 0.01a	0.27 ± 0.06a	0.26 ± 0.02a			

Table S9. Targeted metabolites amino acid and catechins (mg g^{-1}) in leaves of tea plants in response light effect, P effect and their interaction effect.

Metabolites	P level	Light Intensity			Significance		
		FL	ML	LL	light	Plevel	L×P
Amino Acids							
Ser	+P	0.05 ± 0.01d	0.13 ± 0.01a	0.07 ± 0c	$p < 0.001$	$p < 0.001$	$p < 0.001$
	-P	0.09 ± 0.01b	0.06 ± 0.01c	0.13 ± 0.01a			
Gly	+P	0.38 ± 0.15b	0.1 ± 0.03c	0.16 ± 0.04c	$p < 0.001$	$p < 0.001$	ns
	-P	0.16 ± 0.02c	0.57 ± 0.17a	0.35 ± 0.02b			
Thr	+P	0.33 ± 0.11c	0.12 ± 0.05de	0.23 ± 0.02cd	$p < 0.001$	$p < 0.001$	$p < 0.001$
	-P	1.35 ± 0.03a	0.64 ± 0.15b	0.08 ± 0.02e			
Ile	+P	0.67 ± 0.1a	0.28 ± 0.01b	0.08 ± 0.01c	$p < 0.001$	$p < 0.001$	$p < 0.001$
	-P	0.16 ± 0.11c	0.09 ± 0c	0.19 ± 0.05bc			
Val	+P	0.31 ± 0.02a	0.08 ± 0.01c	0.2 ± 0.02b	$p < 0.001$	$p < 0.001$	$p < 0.001$
	-P	0.04 ± 0.02d	0.03 ± 0.01d	0.06 ± 0.03cd			
Phe	+P	1.64 ± 0.06a	0.85 ± 0.01b	0.66 ± 0.12c	$p < 0.001$	$p < 0.001$	$p < 0.001$
	-P	0.68 ± 0.05c	0.32 ± 0.01d	0.05 ± 0.02e			
Ala	+P	0.03 ± 0.01bc	0.01 ± 0cd	0.01 ± 0d	$p < 0.001$	$p < 0.001$	ns
	-P	0.07 ± 0.01a	0.06 ± 0.02a	0.04 ± 0.02b			
Asp	+P	0.26 ± 0.06c	0.63 ± 0.02a	0.21 ± 0.02c	$p < 0.01$	$p < 0.05$	$p < 0.001$
	-P	0.58 ± 0.07a	0.23 ± 0.04c	0.45 ± 0.12b			
Glu	+P	1.68 ± 0.02c	1.81 ± 0.16c	1.7 ± 0.12c	$p < 0.001$	$p < 0.001$	$p < 0.001$
	-P	2.34 ± 0.22b	3.47 ± 0.2a	2.44 ± 0.24b			

Continue on the next page

Table S9. Targeted metabolites amino acid and catechins (mg g^{-1}) in leaves of tea plants in response light effect, P effect and their interaction effect. (*cont.*).

		Light Intensity			Significance		
Metabolites	P level	FL	ML	LL	light	Plevel	L×P
Pro	+P	2.63 ± 0.16b	2 ± 0.11bc	8.35 ± 1.74a	$p < 0.001$	$p < 0.001$	$p < 0.001$
	-P	0.72 ± 0.26cd	0.32 ± 0.09d	2.57 ± 0.55b			
Arg	+P	2.35 ± 0.29b	0.25 ± 0.1d	0.76 ± 0.04c	$p < 0.001$	$p < 0.001$	$p < 0.001$
	-P	0.53 ± 0.13cd	4.91 ± 0.47a	0.42 ± 0.13cd			
Cys	+P	0.28 ± 0.06a	0.32 ± 0.04a	0.13 ± 0.02b	$p < 0.001$	$p < 0.001$	$p < 0.001$
	-P	0.07 ± 0.02c	0.04 ± 0.01c	0.05 ± 0.01c			
His	+P	0.07 ± 0.01c	0.11 ± 0.03c	0.09 ± 0c	$p < 0.001$	$p < 0.001$	$p < 0.001$
	-P	0.25 ± 0.08b	0.38 ± 0.09a	0.07 ± 0.01c			
Leu	+P	0.12 ± 0.02b	0.19 ± 0.02a	0.25 ± 0.03a	$p < 0.01$	$p < 0.001$	ns
	-P	0.24 ± 0.08a	0.12 ± 0.01b	0.07 ± 0.01b			
Lys	+P	0.64 ± 0.04b	0.26 ± 0.08d	0.69 ± 0.07b	$p < 0.001$	$p < 0.001$	$p < 0.001$
	-P	0.44 ± 0.06c	1.24 ± 0.16a	0.32 ± 0.09cd			
Met	+P	1.08 ± 0.1a	0.07 ± 0.01c	0.64 ± 0.11b	$p < 0.001$	$p < 0.001$	$p < 0.001$
	-P	0.09 ± 0.02c	0.68 ± 0.08b	0.06 ± 0.01c			
Thea	+P	22.87 ± 1.03a	11.18 ± 0.57d	13.1 ± 1.37cd	$p < 0.001$	$p < 0.001$	ns
	-P	16.57 ± 0.41b	16.86 ± 2.19b	15.13 ± 0.45bc			
Tyr	+P	0.75 ± 0.07b	0.54 ± 0.27b	0.1 ± 0.03c	$p < 0.001$	$p < 0.001$	$p < 0.001$
	-P	0.48 ± 0.03b	1.47 ± 0.44a	0.5 ± 0.03b			

Continue on the next page

Table S9. Targeted metabolites amino acid and catechins (mg g^{-1}) in leaves of tea plants in response light effect, P effect and their interaction effect. (*cont.*).

Metabolites	P level	Light Intensity FL	ML	LL	Significance light	Plevel	L×P
Catechins							
EGCG	+P	34.38 ± 1.04a	14.79 ± 0.45e	15.37 ± 0.47e	$p < 0.001$	$p < 0.001$	$p < 0.001$
	-P	31.91 ± 0.25b	20.59 ± 0.16d	24.58 ± 0.19c			
C	+P	0.09 ± 0.01b	0.11 ± 0.01a	0.12 ± 0.02a	$p < 0.001$	ns	ns
	-P	0.1 ± 0.01ab	0.12 ± 0.01a	0.11 ± 0.01ab			
CG	+P	0.05 ± 0.03a	0.03 ± 0.01ab	0.03 ± 0ab	$p < 0.001$	ns	ns
	-P	0.04 ± 0a	0.02 ± 0.01b	0.03 ± 0.01ab			
EC	+P	0.16 ± 0.01a	0.16 ± 0.01a	0.16 ± 0.01a	ns	ns	ns
	-P	0.16 ± 0.01a	0.15 ± 0.01a	0.16 ± 0a			
ECG	+P	2.48 ± 0.41a	1.53 ± 0.25b	0.92 ± 0.15c	$p < 0.001$	$p < 0.001$	ns
	-P	1.92 ± 0.38b	0.77 ± 0.15c	0.57 ± 0.12c			
EGC	+P	0.09 ± 0.01b	3.11 ± 0.99a	2.22 ± 0.71a	$p < 0.001$	$p < 0.05$	$p < 0.01$
	-P	0.1 ± 0.01b	2.98 ± 0.7a	0.77 ± 0.18b			
GA	+P	0.32 ± 0.01a	0.32 ± 0.01a	0.32 ± 0.01a	ns	ns	ns
	-P	0.32 ± 0a	0.32 ± 0a	0.32 ± 0a			
GC	+P	0.07 ± 0.03a	0.08 ± 0.04a	0.08 ± 0.04a	ns	ns	ns
	-P	0.07 ± 0.02a	0.05 ± 0.01a	0.08 ± 0.02a			
GCG	+P	0.02 ± 0.01a	0.02 ± 0.01a	0.02 ± 0.01a	ns	ns	ns
	-P	0.02 ± 0.01a	0.02 ± 0a	0.02 ± 0a			

Table S10. Correlation between pathways with phosphorus and biomass due to P effect.

	P levels	Phosphorus		Biomass	
		+P	-P	+P	-P
Phosphorus	+P		0.99***	0.7**	0.5*
	-P	0.99***		0.73***	0.5*
Anthocyanidin and Anthocyanin Biosynthesis	+P	0.95***	0.93***	0.59**	0.58*
	-P	0.89***	0.9***	0.69**	0.44ns
AAP (*amino acid permeases*)	+P	-0.17ns	-0.23ns	-0.39ns	0.08ns
	-P	0.67**	0.61**	0.28ns	0.49*
Isofavone Biosynthesis	+P	-0.61**	-0.57*	-0.16ns	-0.53*
	-P	-0.47*	-0.42ns	-0.07ns	-0.42ns
GSTb (*glutathione S-transferase b*)	+P	-0.7**	-0.66**	-0.28ns	-0.53*
	-P	-0.76***	-0.72***	-0.34ns	-0.54*
Phe, Tyr and Trp biosynthesis	+P	0.01ns	0.06ns	0.38ns	-0.2ns
	-P	-0.45ns	-0.4ns	-0.03ns	-0.4ns
SWEET3 (*bidirectional sugar transporter*)	+P	-0.26ns	-0.3ns	-0.32ns	-0.31ns
	-P	-0.86***	-0.82***	-0.49*	-0.67**
Alanine, Asparate and Glutamate Metabolism	+P	-0.57*	-0.52*	-0.13ns	-0.47ns
	-P	-0.06ns	0ns	0.33ns	-0.25ns
SPX2 (*Pi transport, stress, sensing and signalling*)	+P	0.43ns	0.46ns	0.72***	0.07ns
	-P	-0.74***	-0.71***	-0.25ns	-0.62**

Continue on the next page

Table S10. Correlation between pathways with phosphorus and biomass due to P effect.

	P levels	Phosphorus		Biomass	
		+P	-P	+P	-P
Pentose phosphate pathway	+P	-0.41ns	-0.35ns	0.02ns	-0.41ns
	-P	-0.31ns	-0.25ns	0.06ns	-0.39ns
Biomass	+P	0.7**	0.73***		0.38ns
	-P	0.5*	0.5*	0.38ns	
Inositol phosphate metabolism	+P	0.65**	0.61**	0.23ns	0.5*
	-P	-0.15ns	-0.09ns	0.18ns	-0.33ns

Means with the different letter in the row of the same metabolites content are significantly different.
'***' 0.001 '**' 0.01 '*' 0.05 = significant differences, ns = non-significant differences, between light and P interaction.

Table S11. Correlation between pathways with phosphorus and biomass under different light regimes.

	Light levels	Phosphorus			Biomass		
		FL	ML	LL	FL	ML	LL
Phosphorus	FL		0.96***	0.94***	0.97***	0.89***	0.84***
	ML	0.96***		0.96***	0.93***	0.87***	0.8**
	LL	0.94***	0.96***		0.94***	0.93***	0.8**
Anthocyanidin and Anthocyanin Biosynthesis	FL	0.96***	0.98***	0.96***	0.95***	0.93***	0.85***
	ML	-0.98***	-0.97***	-0.97***	-0.96***	-0.92***	-0.83***
	LL	-0.98***	-0.96***	-0.95***	-0.97***	-0.94***	-0.85***
AAP (*amino acid permeases*)	FL	-0.98***	-0.96***	-0.95***	-0.96***	-0.92***	-0.85***
	ML	-0.98***	-0.95***	-0.93***	-0.94***	-0.91***	-0.84***
	LL	-0.96***	-0.94***	-0.98***	-0.96***	-0.93***	-0.84***
Isofavone Biosynthesis	FL	-0.94***	-0.94***	-0.88***	-0.9***	-0.87***	-0.76**
	ML	-0.75**	-0.69*	-0.65*	-0.7*	-0.65*	-0.58*
	LL	-0.35ns	-0.35ns	-0.33ns	-0.32ns	-0.51ns	-0.42ns
GSTb (*glutathione S-transferase b*)	FL	-0.58ns	-0.44ns	-0.55ns	-0.56ns	-0.65*	-0.65*
	ML	-0.89***	-0.87***	-0.89***	-0.83***	-0.92***	-0.76**
	LL	-0.91***	-0.92***	-0.86***	-0.9***	-0.84***	-0.74**
Phe, Tyr and Trp biosynthesis	FL	-0.7*	-0.53ns	-0.57ns	-0.69*	-0.71**	-0.68*
	ML	-0.95***	-0.88***	-0.85***	-0.89***	-0.79**	-0.78**
	LL	-0.97***	-0.96***	-0.93***	-0.92***	-0.9***	-0.8**
SWEET3 (*bidirectional sugar transporter*)	FL	-0.92***	-0.94***	-0.88***	-0.89***	-0.82**	-0.78**
	ML	-0.95***	-0.89***	-0.92***	-0.93***	-0.93***	-0.88***
	LL	-0.98***	-0.96***	-0.93***	-0.95***	-0.92***	-0.83***

Continue on the next page

Table S11. Correlation between pathways with phosphorus and biomass under different light regimes.

	Light levels	Phosphorus			Biomass		
		FL	ML	LL	FL	ML	LL
Alanine, Asparate and Glutamate Metabolism	FL	0.34ns	0.5ns	0.54ns	0.35ns	0.48ns	0.26ns
	ML	0.97***	0.97***	0.97***	0.96***	0.92***	0.82**
	LL	0.97***	0.97***	0.94***	0.94***	0.91***	0.82**
SPX2 (*Pi transport, stress, sensing and signalling*)	FL	-0.96***	-0.91***	-0.95***	-0.94***	-0.96***	-0.87***
	ML	-0.98***	-0.93***	-0.94***	-0.95***	-0.91***	-0.85***
	LL	-0.98***	-0.93***	-0.92***	-0.94***	-0.92***	-0.84***
Pentose phosphate pathway	FL	0.97***	0.94***	0.95***	0.97***	0.92***	0.87***
	ML	0.95***	0.93***	0.88***	0.89***	0.86***	0.79**
	LL	0.96***	0.96***	0.95***	0.94***	0.94***	0.83***
Biomass	FL	0.97***	0.93***	0.94***		0.92***	0.9***
	ML	0.89***	0.87***	0.93***	0.92***		0.87***
	LL	0.84***	0.8**	0.8**	0.9***	0.87***	
Inositol phosphate metabolism	FL	-0.62*	-0.61*	-0.6*	-0.52ns	-0.49ns	-0.27ns
	ML	-0.96***	-0.94***	-0.97***	-0.97***	-0.95***	-0.85***
	LL	-0.86***	-0.79**	-0.87***	-0.88***	-0.91***	-0.77**

Means with the different letter in the row of the same metabolites content are significantly different.
'***' 0.001 '**' 0.01 '*' 0.05 = significant differences, ns = non-significant differences, between light and P interaction.
